# Transient Receptor Potential Vanilloid 4 Knockdown Decreases Extracellular Matrix Synthesis via Autophagy Suppression in the Rat Intervertebral Disc

**DOI:** 10.1002/jsp2.70046

**Published:** 2025-02-17

**Authors:** Tomoya Matsuo, Yoshiki Takeoka, Takashi Yurube, Takeru Tsujimoto, Yutaro Kanda, Kunihiko Miyazaki, Hiroki Ohnishi, Masao Ryu, Naotoshi Kumagai, Kohei Kuroshima, Yoshiaki Hiranaka, Ryosuke Kuroda, Kenichiro Kakutani

**Affiliations:** ^1^ Department of Orthopaedic Surgery Kobe University Graduate School of Medicine Kobe Japan

**Keywords:** autophagy, extracellular matrix, intervertebral disc, nucleus pulposus cell, RNA interference (RNAi), spine, transient receptor potential vanilloid 4 (TRPV4)

## Abstract

**Background:**

Transient receptor potential vanilloid 4 (TRPV4) has been identified as a Ca^2+^‐permeable channel and is activated under physiological mechanical stimulation in disc nucleus pulposus (NP) cells. Meanwhile, the Ca^2+^‐dependent AMP‐activated protein kinase (AMPK)/mTOR pathway activates autophagy in notochordal cells. We hypothesized that TRPV4 is involved in the maintenance of intradiscal homeostasis via autophagy. Our objective was to elucidate the role of TRPV4 in extracellular matrix (ECM) metabolism and autophagy in the rat intervertebral disc through a loss‐of‐function study with the RNA interference (RNAi) technique.

**Methods:**

In vitro study: Small interfering RNA (siRNA) was applied to knockdown TRPV4 by the reverse transfection method in rat disc NP cells. Expression of TRPV4, AMPK/mTOR pathway‐related markers, and autophagy markers were measured by Western blotting (WB). Next, ECM metabolism was assessed under serum starvation and/or proinflammatory interleukin‐1 beta (IL‐1β) stimulation. In vivo study: TRPV4 and control siRNAs were injected into rat discs. To confirm in vivo transfection, WB for TRPV4 was conducted in rat disc NP‐tissue protein extracts 2, 28, and 56 days after injection. Furthermore, 24‐h temporary static compression‐induced disruption of TRPV4 siRNA‐injected discs was observed by radiography, histomorphology, and immunofluorescence.

**Results:**

In vitro study: In disc cells, three different TRPV4 siRNAs consistently suppressed autophagy with TRPV4 protein knockdown (mean 33.2% [95% CI: −50.8, −15.5], 44.1% [−61.7, −26.4], 58.3% [−76.0, −40.7]). ECM metabolism was significantly suppressed by TRPV4 RNAi under proinflammatory IL‐1β stimulation. In vivo study: The WB displayed sustained decreases in TRPV4 protein expression 2, 28, and 56 days after injection. Under the loaded condition, TRPV4 siRNA‐injected discs presented radiographic height loss ([−31.7, −7.75]), histomorphological damage ([0.300, 4.70]), and immunofluorescent suppression of autophagy ([1.61, 20.5]) and ECM metabolism ([−25.2, −6.41]) compared to control siRNA‐injected discs at 56 days.

**Conclusions:**

The TRPV4 could be a therapeutic target for intervertebral disc diseases via modulating autophagy.

## Graphical abstract

We conducted in vitro and in vivo TRPV4 RNA interference study in the rat intervertebral disc cells and tissues. In vitro, TRPV4 knockdown decreased extracellular matrix synthesis, cell viability, and autophagy activity. In vivo, TRPV4 knockdown showed radiographic intervertebral disc height loss, histomorphological disc degeneration, and immunofluorescent autophagy suppression.

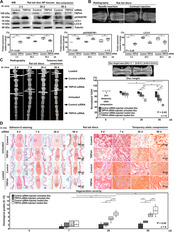

## 1 Introduction

Back pain is a global health, workforce, and socioeconomic problem, [1] and intervertebral disc degeneration is one of the major independent risk factors [2]. The intervertebral disc is the largest avascular low‐nutrient organ in the body, comprising the gelatinous nucleus pulposus (NP) confined by the collagenous annulus fibrosus (AF) and cartilaginous endplates, and supports compressive loading and facilitating multidimensional spinal movement [3]. Recently, disc degeneration has been suggested to be associated with a series of physiopathologic changes in NP cells, especially autophagy [4]. Autophagy, an intracellular clearance mechanism in which cells degrade and recycle their proteins and organelles, is negatively regulated by the mammalian target of rapamycin (mTOR) signaling pathway [5]. The mTOR exists in two complexes: mTOR complex 1 (mTORC1) containing the regulatory‐associated protein of mTOR (RAPTOR) and mTOR complex 2 (mTORC2) containing the rapamycin‐insensitive companion of mTOR (RICTOR) [5]. Downstream mTORC1 effectors including p70/ribosomal S6 kinase (p70/S6K) regulate cell proliferation, messenger RNA (mRNA) translation, and protein synthesis [5]. In rat notochordal cells, autophagy develops by osmotic stimulation via Ca‐ion‐dependent AMP‐activated protein kinase (AMPK)/mTOR pathway [6]. Moreover, disc degeneration is biochemically characterized by ECM degradation [7]. ECM metabolism is regulated by the balance between catabolic enzymes, primarily ECM metalloproteinases (MMPs), and their anticatabolic inhibitors, tissue inhibitors of metalloproteinases (TIMPs) [8]. Increased MMPs relative to TIMPs are often observed in human clinical [9, 10, 11] and rodent experimental disc degeneration [11, 12, 13], leading to degraded matrix components including proteoglycans, principally Aggrecan, and collagens, predominantly types II (COL2A1) in the NP [7]. Our human disc NP cell study through posttranscriptional gene silencing by RNA interference (RNAi) has found protective effects of specific mTORC1 suppression against matrix catabolism with enhanced autophagy [14]. Our rat disc study has found autophagy‐related gene 5 (Atg5)‐dependent autophagy is involved in the maintenance of disc homeostasis [15]. These indicated the relationship among autophagy, homeostasis, and extracellular matrix (ECM) in the intervertebral disc.

Previous studies demonstrated that disc NP cells and their ECM are stimulated by the physiological range of mechanical loading with transient receptor potential (TRP) vanilloid 4 (TRPV4) activation in the bovine and the human [16, 17], and abnormal loading decreases ECM expression, resulting in disc degeneration in the rat [18]. In articular cartilage, TRPV4 activation with an agonist stimulates anabolic turnover and suppresses catabolic turnover, leading to ECM component accumulation [19]. The TRPV4 is included in the TRP channel family, which are nonselective Ca‐ion‐permeable transmembrane channel‐linked receptors. They attract particular interest in the intervertebral disc research field because they sense physicochemical changes observed during disc degeneration: pH, oxidative stress, and mechanical loading [20]. The TRP superfamily is composed of six subfamilies in mammals: TRPC (canonical), TRPV (vanilloid), TRPM (melastatin), TRPP (polycystin), TRPML (mucolipin), and TRPA (ankyrin) [20, 21]. While the expression and functions of TRP channels in the intervertebral disc have been reported, the highest expression among TRP channels in the disc is TRPV4 [22, 23, 24].

**FIGURE 1:**
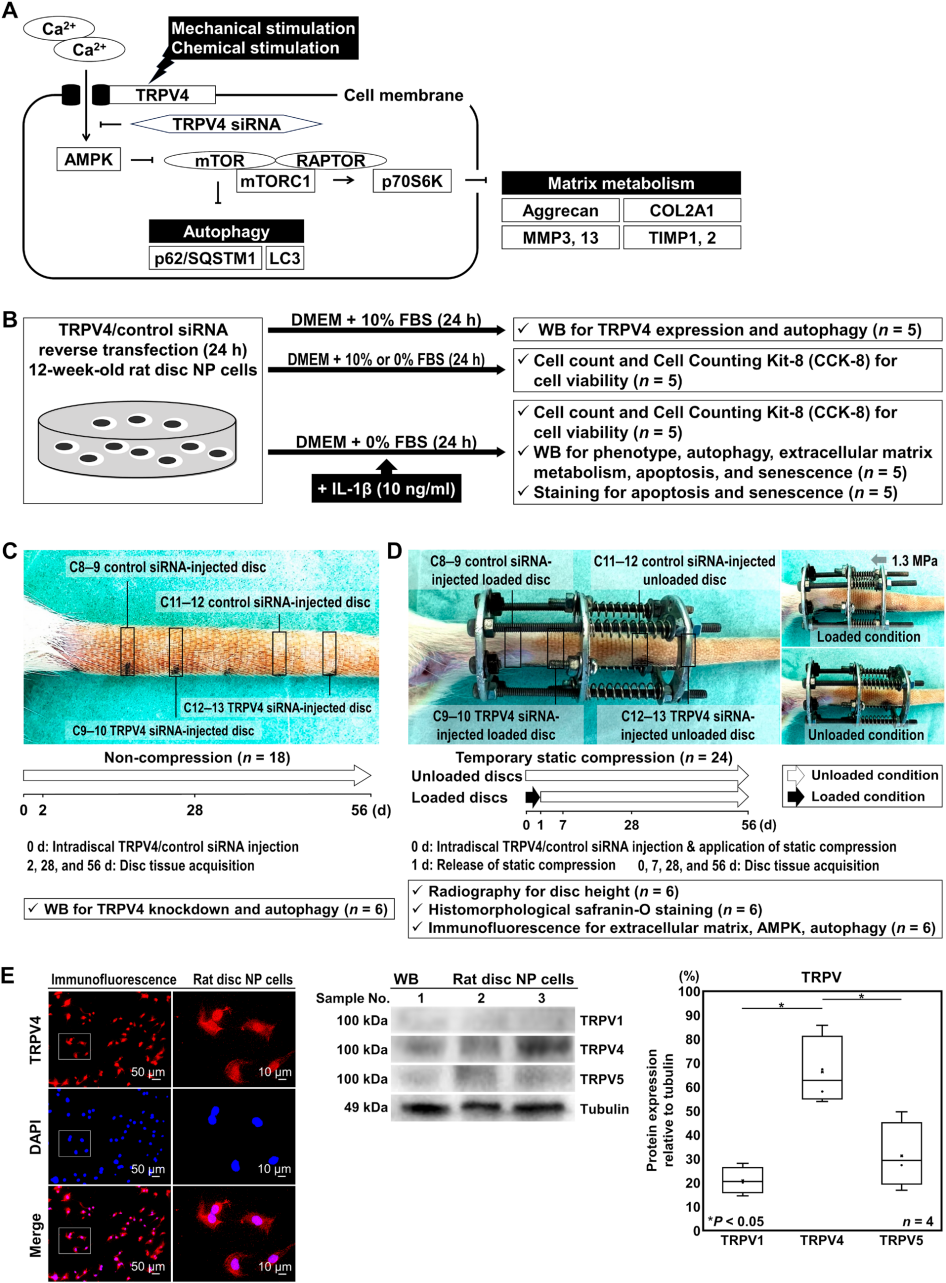
Schematic illustration of disc cellular autophagy and extracellular matrix (ECM) metabolism under mechanical and chemical stimulation, in vitro and in vivo experimental regimens, and confirmation of intradiscal TRPV4 expression. (A) Schematic diagram of the mechanism involved in the protective role of autophagy and ECM metabolism via Ca ion‐dependent AMPK/mTOR pathway under mechanical or chemical stimulation. By TRPV4 siRNA, Ca^2+^ influx is blocked and AMPK is suppressed. Then, mTORC1 and p70S6K are activated, resulting in suppression of autophagy and ECM metabolism. (B) Schematic illustration of the in vitro study design and the number of samples. First passage, ~80%‐confluent, monolayer disc NP cells from 12‐week‐old male rats (*n* = 20) were reverse transfected with siRNAs targeting TRPV4 or nonspecific control in DMEM with 10% FBS under 2% O_2_ for 24 h and analyzed by WB for TRPV4 knockdown and autophagy (*n* = 5; by cell count and CCK‐8 for cell viability after 24‐h serum deprivation in DMEM with 0% or 10% FBS (*n* = 5); by cell count, CCK‐8 for cell viability, WB for the phenotype, ECM metabolism, apoptosis, and senescence, staining for apoptosis and senescence after 24‐h serum withdrawal and proinflammatory stimulation in DMEM with 0% FBS and 10‐ng/ml IL‐1β (each *n* = 5). (C) Schematic illustration of the in vivo siRNA transfection study design and the number of samples. In rat tails (*n* = 18), control siRNA was injected using a 33‐gauge needle into C8–9 and C11–12 discs, while TRPV4 siRNA was injected into C9–10 and C12–13 discs. At 2–56 d, tissues of C8–9 control siRNA‐injected and C9–10 TRPV4 siRNA‐injected discs and C11–12 control siRNA‐injected and C12–13 TRPV4 siRNA‐injected discs were acquired and analyzed by WB for TRPV4 knockdown and autophagy, respectively (*n* = 6/time point). (D) Schematic illustration of the in vivo temporary static compression study design and the number of samples. In rat tails (*n* = 24), an Ilizarov‐type apparatus with springs was affixed between C8 and 10. Control siRNA was injected into C8–9 and C11–12 discs, whereas TRPV4 siRNA was injected into C9–10 and C12–13 discs. Then, 1.3‐MPa axial force was applied to C8–9 and C9–10 discs for 24 h and subsequently released. At 0–56 d, tissues of C8–9 control siRNA‐injected loaded, C9–10 TRPV4 siRNA‐injected loaded, C11–12 control siRNA‐injected unloaded, and C12–13 TRPV4 siRNA‐injected unloaded discs were acquired following radiography for the height and analyzed by histomorphological safranin‐O staining and immunofluorescence for ECM, AMPK, and autophagy (*n* = 6/time point). (E) Confirmation of intradiscal TRPV4 expression. Immunofluorescence showed TRPV4 expression in the NP cells. WB for TRPV1, 4, 5, and loading control tubulin of total protein extracts from rat disc NP cells is shown and changes in relative protein expression of TRPV1, 4, and 5 normalized to tubulin are semiquantified. Data are presented with dot and box plots (*n* = 4). One‐way repeated measures ANOVA with the Tukey–Kramer post hoc test was used. Immunoblots shown are representative of experiments with similar results.

Based on these findings, we hypothesized that TRPV4 contributes to intradiscal homeostasis via the Ca‐ion‐dependent AMPK/mTOR pathway and autophagy (Figure 1A). Our objective was to elucidate the role of TRPV4 in ECM metabolism and autophagy in the rat intervertebral disc through a loss‐of‐function study with the RNAi technique.

## 2 Materials and Methods

### 2.1 Ethics Statement

All experimental procedures were performed in accordance with the Institutional Animal Care and Use Committee at the authors' institution.

### 2.2 Antibodies and Reagents

The antibodies and reagents used are listed in Table S1.

### 2.3 Cells

Sixty‐two 12‐week‐old male Sprague Dawley rats (mean 475.5 g [95% confidence interval (CI): 470.5, 482.0]) purchased from CLEA Japan (Tokyo, Japan) were randomly applied to in vitro and in vivo experiments. Coccygeal (C) discs from 20 rats (472.5 g [463.4, 485.6]) were dissected into the NP and AF after euthanasia. Rat disc NP tissues were digested in 1% penicillin/streptomycin‐supplemented Dulbecco's modified Eagle's medium (DMEM) with 10% fetal bovine serum (FBS) and 0.114% collagenase type 2 for 1 h at 37°C. Isolated disc NP cells were grown to ~80% confluence as a monolayer in DMEM with 10% FBS under 2% O_2_ at 37°C. To retain the phenotype, only first‐passage cells were used [25, 26, 27, 28].

The density of randomly distributed seeding cells (*n* = 25) was 5.0 × 10^3^/well (96‐well plate) for viability, 1.5 × 10^5^/well (6‐well plate) for protein extraction, and 1.2 × 10^4^/well (8‐well chamber) for staining. In respective experiments, 5 cell samples from 5 different animals (each *n* = 5) were tested based on the literature [25, 26, 27, 28].

First of all, Western blotting (WB) for TRPV1, 4, and 5 which were reported to be highly expressed in the intervertebral discs was performed [23, 29] (Figure 1E).

After 24‐h RNAi treatment in 10% FBS‐supplemented DMEM, cells were applied to WB for disc NP phenotype, TRPV4 knockdown, and autophagy. Additionally, to simulate clinically relevant disease conditions of serum deprivation and/or inflammation, cells were cultured for 24 h in DMEM with 10% FBS, with 0% FBS, or with 0% FBS and 10‐ng/ml interleukin‐1 beta (IL‐1β), a proinflammatory cytokine related to the pathogenesis [27, 30], and severity of disc degeneration [31].

Then, cell viability was assessed using the Cell Counting Kit‐8 (CCK‐8) and microscopic cell count was also performed. WB for ECM metabolism, apoptosis, and senescence, apoptotic terminal deoxynucleotidyl transferase dUTP nick end labeling (TUNEL) staining, and senescence‐associated beta‐galactosidase (SA‐β‐gal) staining were conducted (Figure 1B).

### 2.4 Animals and Surgical Procedures

Forty‐two rats (477.1 g [471.3, 482.9]) were used. Under general anesthesia, 2‐ml TRPV4 siRNA solution was injected using a 33‐gauge needle at the disc center through a 5‐mm longitudinal skin incision [32, 33]. At 2–56 days, C8–9 control siRNA‐injected and C9–10 TRPV4 siRNA‐injected disc and C11–12 control siRNA‐injected and C12–13 TRPV4 siRNA‐injected disc tissues were acquired and analyzed by WB for TRPV4 knockdown and autophagy, respectively (Figure 1C).

A rat tail temporary static compression model was used to reproduce mechanical stress‐induced disc degenerative changes [12, 13, 18, 25, 34]. Under general anesthesia, an Ilizarov‐type apparatus with springs was attached between the C8 and C10 vertebrae of rat tails (*n* = 24). Control and TRPV4 siRNA–Invivofectamine 3.0 reagent complexes were injected into C8–9 and C11–12 discs and C9–10 and C12–13 discs, respectively. Then, 1.3‐MPa axial force, corresponding to a disc loading force produced by lifting a moderate weight in the human lumbar spine, [1, 35] was applied to C8–9 and C9–10 discs for 24 h and subsequently released. At 0–56 days, C8–9 control siRNA‐injected loaded, C9–10 TRPV4 siRNA‐injected loaded, C11–12 control siRNA‐injected unloaded, and C12–13 TRPV4 siRNA‐injected unloaded disc tissues were acquired after radiographic imaging and analyzed by histomorphological safranin‐O staining and immunofluorescence (Figure 1D).

Sample size (*n* = 5 in vitro and 6 in vivo/time point) was based on the literature [12, 13, 18, 25, 32, 33, 34]. Rats were fed separately in a specific pathogen‐free housing cage with freely available food and water. The room had a controlled 12‐h light/dark cycle, temperature (23°C ± 2°C), and humidity (55% ± 5%). Humane endpoints, for example, ≥ 20% weight loss and behavioral changes, were determined.

### 2.5 RNAi

The RNAi was performed using small interfering RNAs (siRNAs) to knockdown TRPV4 with the reverse transfection method, allowing high transfection efficiency [36]. In vitro, three different TRPV4 siRNAs (Table S2) were used to minimize off‐target effects. A nontargeting siRNA was used as a negative control. Cells in 10% FBS‐supplemented DMEM were added to each siRNA with Lipofectamine RNAiMAX transfection reagent diluted in Opti‐Minimal Essential Medium I and then cultured for 24 h. Applied amounts of siRNAs were 60 (6‐well plate), 4.8 (8‐well chamber), and 2 (96‐well plate) pmol/well. In vivo, TRPV4 or control siRNA with Invivofectamine 3.0 reagent (the final concentration of 1.5‐pmol/L) was prepared.

### 2.6 Cell Viability Assay

In vitro, cell viability was assessed by CCK‐8 dehydrogenase activity, the absorbance of which (450 nm) was measured by the Model 680 microplate reader. In addition, images were obtained with the BZ‐X700 microscope. The number of adherent cells was counted in duplicated four random low‐power fields (×100) (LPFs) using the ImageJ software (https://imagej.nih.gov/ij/).

### 2.7 Protein Extraction, Sodium Dodecyl Sulfate (SDS) ‐Polyacrylamide Gel Electrophoresis (PAGE), and WB

Cells were scraped off on ice in 3‐(N‐morpholino)propane‐sulfonic acid buffer containing protease and phosphatase inhibitors. Harvested tissues were homogenized using the MS‐100R bead‐beating disrupter for 30 s twice at 4°C in the T‐PER tissue protein extraction reagent with protease and phosphatase inhibitors. Soluble proteins were collected after 20 000‐ × *g* centrifugation for 15 min at 4°C. Samples were stored at −80°C. Protein concentration was determined by the bicinchoninic acid assay.

Equal 30‐μg amounts of protein were mixed with the SDS–PAGE sample buffer, boiled for 5 min, and resolved on a 7.5%–15.0% polyacrylamide gel. Separated proteins in the tris(hydroxymethyl) aminomethane–glycine–SDS buffer system were transblotted to a polyvinylidene difluoride membrane and probed with primary antibodies for 12 h at 4°C (1:200–1:1000 dilution) followed by secondary antibodies (1:400 dilution) for 1 h at room temperature. Signals were visualized by enhanced chemiluminescence. Images were obtained using the Chemilumino analyzer LAS‐3000 mini. Band intensity was quantified using ImageJ.

In vitro, WB was designed to analyze the intracellular expression of notochordal Brachyury and CD24 [30], anabolic Aggrecan, COL2A1 [37], anti‐catabolic TIMPs, catabolic MMPs, TRPV4, AMPK, mTOR, p70S6K, autophagy‐related p62/SQSTM1 and LC3 [38], apoptosis‐related poly (ADP‐ribose) polymerase (PARP) and cleaved caspase‐9 [39], and senescence‐related p53, p21/CIP1, and p16/INK4a [40] in total cell or tissue protein extracts. Among these, Aggrecan was analyzed following deglycosylation by chondroitinase ABC and endo‐β‐galactosidase [41]. In vivo, WB was designed to analyze TRPV4 knockdown and autophagy activity. Protein expression was normalized to loading control tubulin and shown as the relative percentage of control.

### 2.8 Paraffin‐Embedded Tissue Preparation

In vivo, functional rat caudal spinal units (vertebral body–disc–vertebral body) were obtained after euthanasia. Then, the specimens were fixed en‐bloc with 4% paraformaldehyde for 1 day, decalcified in 10% ethylenediaminetetraacetic acid for 7 days, embedded in paraffin, and cut mid‐sagittal into 7‐μm sections for histomorphological staining and immunofluorescence.

### 2.9 TUNEL Staining

In vitro, cells were fixed with 4% paraformaldehyde for 10 min and applied to fluorescein‐labeled TUNEL staining for apoptotic fragmented DNA detection with 4′,6‐diamidino‐2‐phenylindole (DAPI) for counterstaining [37, 42]. Under the BZ‐X700 microscope and ImageJ, the percentage of TUNEL‐positive cells was calculated relative to the total number of DAPI‐positive cells in duplicated four random LPFs.

### 2.10 SA‐β‐Gal Staining

In vitro, senescent cells were identified by cytochemical SA‐β‐gal staining at pH 6.0 [43]. The percentage of SA‐β‐gal‐positive cells was calculated in duplicated four random LPFs.

### 2.11 Immunofluorescence

In vitro, multicolor immunofluorescence was performed to assess disc cellular TRPV4 expression and in vivo TRPV4, ECM, AMPK, and autophagy. After antigen retrieval, permeabilization, and blocking, fixed cells and disc tissue sections were incubated with TRPV4, COL2A1, Brachyury, p‐AMPK, p62/SQSTM1, and LC3‐II primary antibodies (all, 1:100 dilution) for 12 h at 4°C and subsequently with Alexa Fluor 488, 568, and 647 secondary antibodies (1:200 dilution) and DAPI for 1 h at room temperature. The percentage of positive cells was calculated relative to DAPI‐positive cells in duplicated four random LPFs.

We employed a rat IgG control antibody as a negative control to confirm the specificity of our immunohistochemistry staining. These control experiments demonstrated that the observed staining was specific to our target antigen.

### 2.12 Radiography

In vivo, lateral radiographs were taken using a VPX‐30E system and IXFR film (exposure time, 40 s; distance, 40 cm; current, 3 mA; voltage, 35 kV). Disc height was measured using ImageJ twice at a 1‐week interval by each of two investigators blinded to this study, normalized to adjacent vertebral body heights as the disc height index (DHI), shown as the percent of preoperative DHI (%DHI = [postoperative DHI/preoperative DHI] × 100), and further normalized to the intact disc as the normalized %DHI (normalized %DHI = [experimental %DHI/intact %DHI] × 100) [44].

### 2.13 Histomorphology

In vivo, safranin‐O, fast green, and hematoxylin staining were performed to demonstrate disc tissue morphological disruption. Histopathological grade, from 0 (nondegenerated) to 16 (severely degenerated) [45], was assessed in duplicate twice by each of two blinded investigators, and the median scores were used for evaluation.

### 2.14 Statistical Analysis

Data are presented as the mean [95% CI: lower limit, upper limit] in the text and box plots in the graphs. With the normality assumption, multiway repeated measure analysis of variance (ANOVA) with the Tukey–Kramer post hoc test was used to assess the effects of “treatment,” “experimental condition,” and “time” on in vitro cell viability, cell count, WB, TUNEL staining, and immunofluorescence, on in vivo WB, and on in vivo radiography, histomorphology, and immunofluorescence, respectively. Statistical analysis was performed using IBM SPSS Statistics 23.0 (IBM, Armonk, NY).

## 3 Results

### 3.1 Confirmation of TRPV4 Expression and Comparison of TRPV1, TRPV4, and TRPV5 Expression in Rat Intervertebral Discs

First, we confirmed TRPV4 expression in the NP cells of the rat intervertebral disc by immunofluorescence, then assessed TRPV1, TRPV4, and TRPV5 expression by WB. In the rat discs, TRPV4 expressed 63.5% relative to tubulin, which was approximately 2.68 times higher than TRPV1 (23.7%, [19.7, 59.9]) and 2.03 times higher than TRPV5 (36.4%, [12.1, 52.4]) (Figure 1E).

### 3.2 In Vitro TRPV4 Knockdown Suppresses Autophagy and ECM Synthesis in Rat Disc NP Cells

**FIGURE 2:**
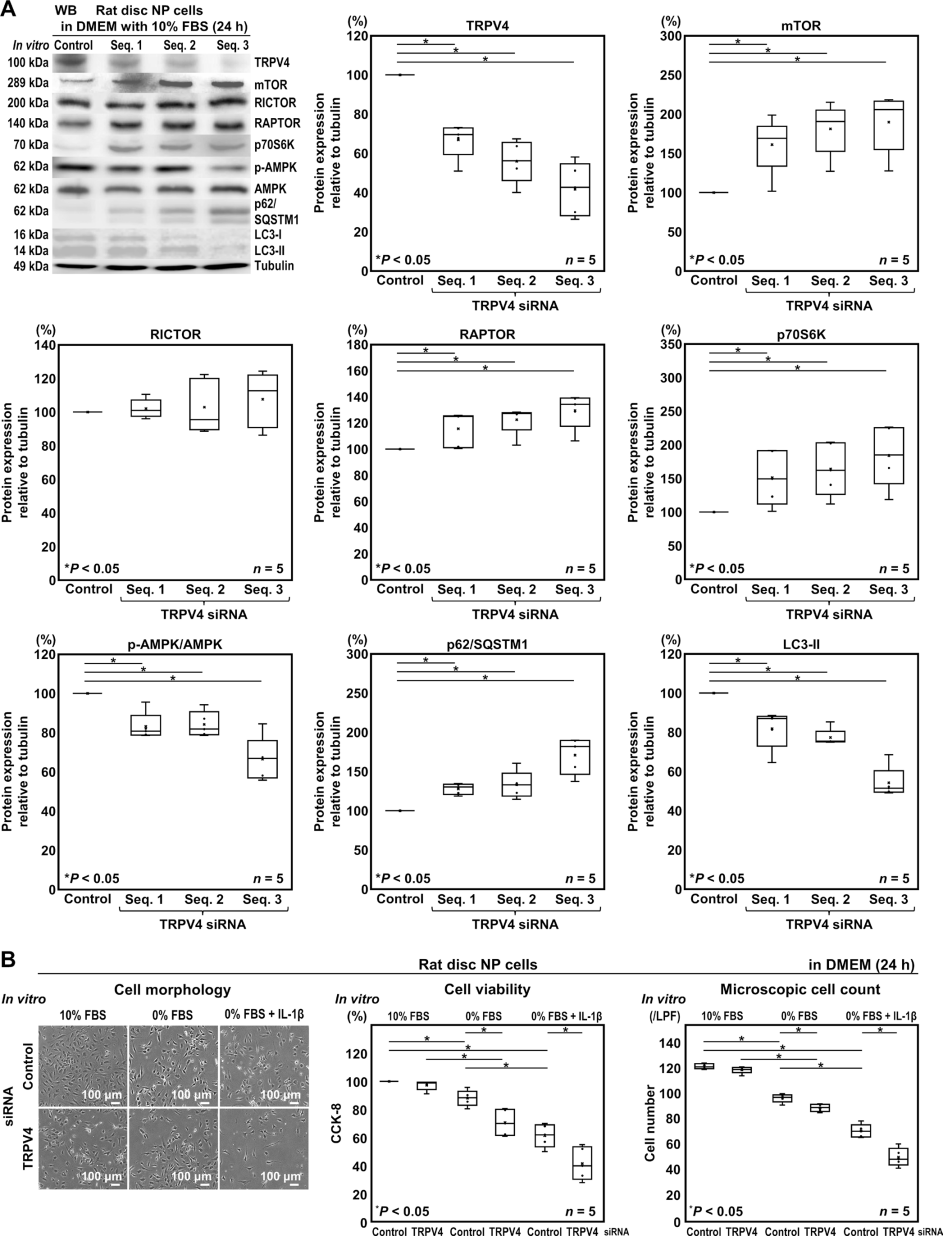
In vitro TRPV4 knockdown suppresses autophagy in rat disc NP cells. (A) WB for TRPV4, mTOR, RICTOR, RAPTOR, p70S6K, AMPK (p‐AMPK/AMPK), p62/SQSTM1, LC3, and loading control tubulin of total protein extracts from rat disc NP cells after TRPV4 or control siRNA transfection in DMEM with 10% FBS for 24 h. Changes in relative protein expression of TRPV4, mTOR, RAPTOR, p70S6K, AMPK, p62/SQSTM1, LC3‐II, and normalized to tubulin are shown. (B) Changes in morphological appearance, CCK‐8‐based cell viability, and cell count of rat disc NP cells after TRPV4 or control siRNA transfection in DMEM with 10% FBS for 24 h followed by in DMEM with 10% FBS, with 0% FBS, or with 0% FBS and 10‐ng/ml IL‐1β for 24 h. Cell count was performed in respective four random LPFs of duplicates. In (A, B), data are presented with box plots (*n* = 5). One‐way repeated measures ANOVA with the Tukey–Kramer post hoc test was used. Immunoblots and cellular images shown are representative of experiments with similar results.

We assessed whether TRPV4 RNAi could effectively knockdown the corresponding protein. A significant decrease in TRPV4 expression was seen following three TRPV4‐siRNA transfection (sequence 1, mean 33.2% knockdown [95% CI: −50.8, −15.5]; sequence 2, 44.1% knockdown [−61.7, −26.4]; and sequence 3, 58.3% knockdown [−76.0, −40.7]). Furthermore, we assessed TRPV4‐RNAi effects on autophagy via the AMPK/mTOR pathway. In WB, sequence‐3 TRPV4 siRNA exhibited the highest effect, which significantly decreased p‐AMPK/AMPK (66.5%, [−46.9, −20.1]), increased mTOR (189.8%, [34.2, 145.3]) and p70S6K (184.0%, [63.8, 247.4]), increased p62/SQSTM1 (170.8%, [10.5, 64.6]), and decreased LC3‐II (54.3%, [−58.1, −33.4]). In addition, the sequence‐3 TRPV4 siRNA treatment significantly increased RAPTOR (189.8%, [8.62, 50.2]), whereas it did not affect RICTOR (107.6%, [−13.5, 28.8]) (Figure 2A). These results indicated successful TRPV4 knockdown‐mediated autophagy suppression via AMPK/mTOR pathway, particularly through mTORC1. In subsequent experiments, the sequence‐3 TRPV4 siRNA with the highest knockdown efficiency was used.

Second, to understand TRPV4 RNAi‐modified disc cellular physiology, we assessed cell viability by CCK‐8 and conducted microscopic cell counting. They showed a decreasing tendency by treatment of TRPV4 siRNA in 10% FBS‐supplemented DMEM (CCK‐8, 100% vs. 97.2% [−14.1, 8.42]; cell number, 122.4/LPF vs. 119.6/LPF [−9.46, 3.86]) and a significant decrease in serum‐free DMEM (CCK‐8, 88.0% vs. 70.7% [−60.6, −28.5]; cell number, 97.4/LPF vs. 89.8/LPF [−14.3, −0.940]). Cell viability and cell number further decreased by TRPV4 knockdown under IL‐1β stimulation (CCK‐8, 61.4% vs. 41.2% [−34.4, −5.2]; cell number, 72.2/LPF vs. 51.6/LPF [−29.0, −12.2]). These results indicated that TRPV4 knockdown reduced the cell viability and cell number further under serum deprivation and inflammation (Figure 2B).

**FIGURE 3:**
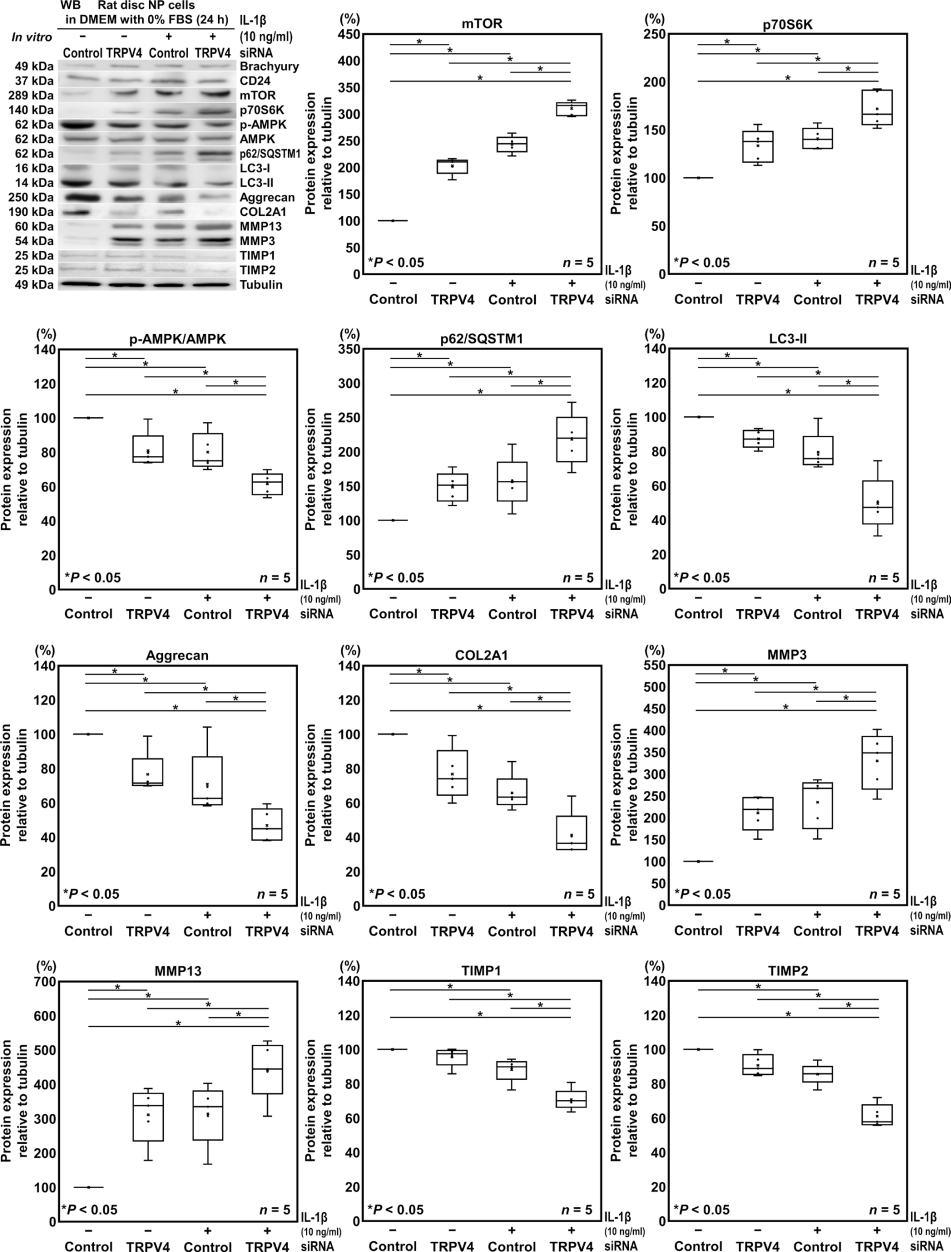
In vitro TRPV4 knockdown suppresses ECM synthesis in rat disc NP cells. Western blotting for phenotypic Brachyury and CD24, AMPK/mTOR pathway markers (mTOR, p70S6K, and AMPK), autophagy (p62/SQSTM1 and LC3‐II), and ECM metabolism (COL2A1, Aggrecan, catabolic MMP3 and MMP13, and anticatabolic TIMP1 and TIMP2) in supernatant protein extracts from rat disc NP cells after 24‐h transfection of TRPV4 or control siRNA in DMEM with 10% FBS followed by 24‐h culture in serum‐free DMEM with 10‐ng/ml IL‐1β. Changes in relative protein expression of mTOR, p70S6K, AMPK, p62/SQSTM1, LC3‐II, COL2A1, Aggrecan, MMP3, MMP13, TIMP1, and TIMP2 normalized to tubulin are shown. Data are presented with box plots (*n* = 5). One‐way repeated measures ANOVA with the Tukey–Kramer post hoc test was used. Immunoblots shown are representative of experiments with similar results.

Third, we assessed TRPV4‐RNAi effects on disc ECM metabolism. Notochordal marker expression was evaluated to validate rat disc NP phenotype. In WB, protein extracts from tested samples all showed positive expression of notochord‐related Brachyury and CD24. The WB demonstrated that TRPV4‐siRNA treatment significantly decreased Aggrecan (76.6%, [−45.7, −1.01]) and COL2A1 (76.7%, [−43.6, −2.93]), and increased MMP3 (211.1%, [24.9, 197.4]) and MMP13 (311.2%, [77.5, 344.9]) protein expression, while it did not affect TIMP1 (95.7%, [−14.2, 5.5]) and TIMP2 (86.3%, [−19.3, 0.756]) expression. Proinflammatory IL‐1β stimulation resulted in significant downregulation of the AMPK/mTOR pathway (p‐AMPK/AMPK, 80.1%, [−34.9, −5.0]; mTOR, 242.9%, [119.7, 166.1]; p70S6K, 140.8%, [15.9, 65.7]), autophagy (p62/SQSTM1, 156.3%, [5.22, 107.4]; LC3‐II, 79.5%, [−38.7, −2.29]), and ECM metabolism (Aggrecan, 70.8%, [−51.6, −6.82]; COL2A1, 65.8%, [−54.5, −13.9]; TIMP1, 88.2%, [−21.6, −1.93]; TIMP2, 85.6%, [−24.4, −4.35]; MMP3, 235.5%, [49.2, 221.8]; MMP13, 314.2%, [80.5, 347.9]). This catabolic effect by autophagy suppression via the AMPK/mTOR pathway was further enhanced by TRPV4 RNAi under IL‐1β stimulation (Aggrecan, 46.9%, [−75.5, −30.8]; COL2A1, 41.3%, [−79.0, −38.4]; MMP3, 330.2%, [144.0, 316.5]; MMP13, 443.0%, [209.3, 476.7]; TIMP1, 70.8%, [−39.1, −19.3]; TIMP2, 61.1%, [−48.9, −28.8]) (Figure 3).

### 3.3 In Vitro TRPV4 Knockdown Increases the Incidence of Apoptosis and Senescence in Rat Disc NP Cells

**FIGURE 4:**
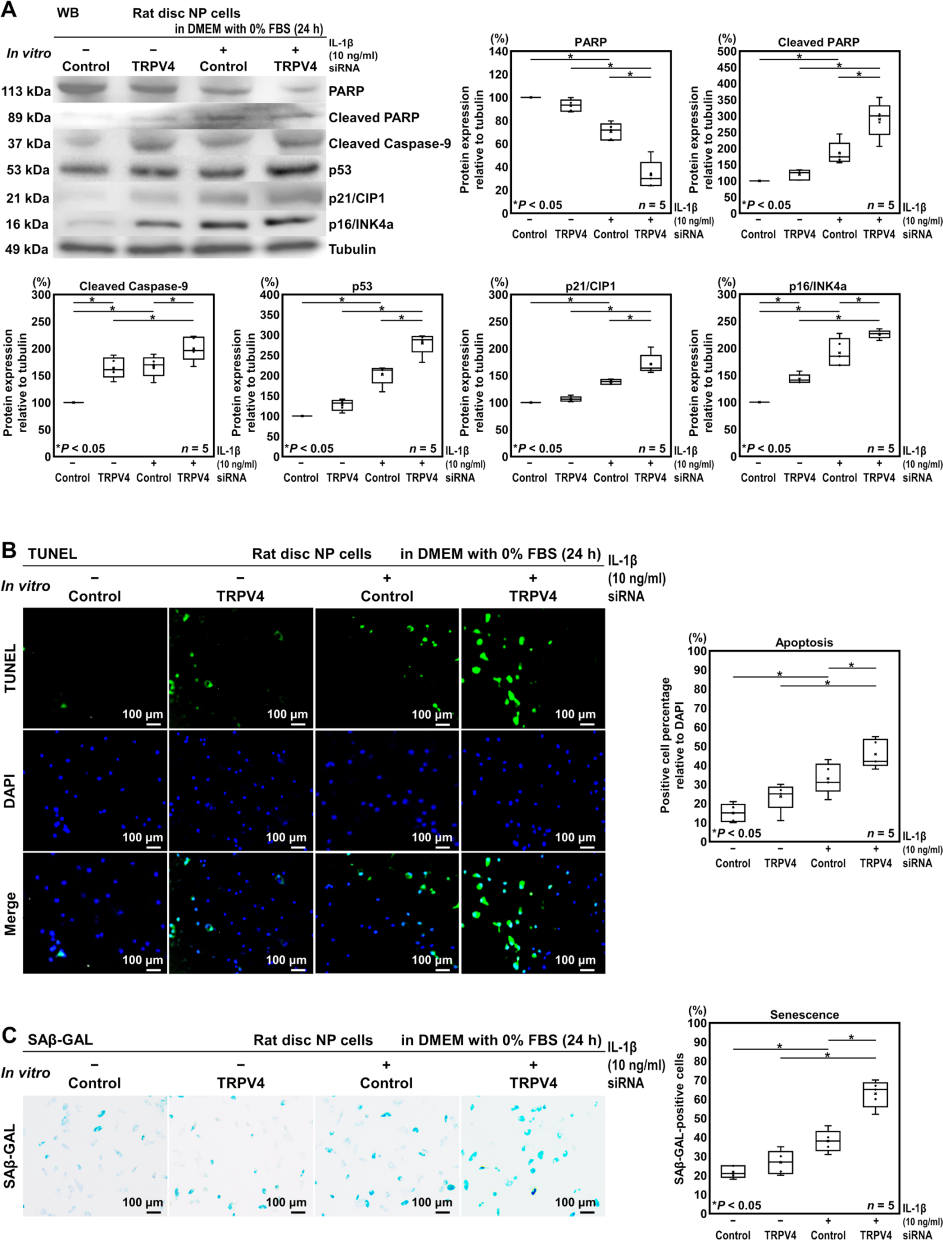
In vitro TRPV4 knockdown increases the incidence of apoptosis and senescence in rat disc NP cells. (A) WB for apoptotic PARP, cleaved PARP, and cleaved caspase‐9, senescent p53, p21/CIP1, and p16/INK4a, and loading control tubulin of total protein extracts from rat disc NP cells after TRPV4 or control siRNA transfection in DMEM with 10% FBS for 24 h followed by in DMEM with 0% FBS and 10‐ng/ml IL‐1β for 24 h. Changes in relative protein expression of PARP, cleaved PARP, cleaved caspase‐9, p53, p21/CIP1, and p16/INK4a normalized to tubulin are shown. (B) Immunofluorescence for apoptotic TUNEL (green), nuclear DAPI (blue), and merged signals of rat disc NP cells after TRPV4 or control siRNA transfection in DMEM with 10% FBS for 24 h followed by in DMEM with 0% FBS and 10‐ng/ml IL‐1β for 24 h. Changes in the percentage of TUNEL‐positive cells relative to DAPI‐positive cells are shown. (C) SA‐β‐gal staining of rat disc NP cells after TRPV4 or control siRNA transfection in DMEM with 10% FBS for 24 h followed by in DMEM with 0% FBS and 10‐ng/ml IL‐1β for 24 h. Changes in the percentage of SA‐β‐gal‐positive cells relative to total cells are shown. In (A), data are presented with box plots (*n* = 5). In (B, C), cell count was performed in respective four random LPFs of duplicates. Data are presented with box plots (*n* = 5). One‐way repeated measures ANOVA with the Tukey–Kramer post hoc test was used. Immunoblots and immunofluorescent and cytochemical images shown are representative of experiments with similar results.

The TRPV4 knockdown significantly increased apoptotic cleaved caspase‐9 (164.0%, [32.0, 96.0]) and senescent p16/INK4a (143.1%, [17.6, 68.5]) without proinflammatory IL‐1β stimulation, while the other markers did not change significantly (PARP, 92.9%, [−20.5, 6.34]; cleaved PARP, 119.2%, [−41.2, 79.6]; p53, 127.5%, [−7.48, 62.6]; p21/CIP1, 106.3%, [−4.93, 17.6]). The IL‐1β stimulation induced significant downregulation of apoptosis‐related PARP (70.5%, [−42.9, −16.1]) and upregulation of apoptotic cleaved PARP (185.1%, [24.7, 145.5]) and cleaved caspase‐9 (167.0%, [35.0, 98.9]) and senescent p53 (202.5%, [67.5, 137.5]), p21/CIP1 (138.2%, [27.0, 49.5]) and p16/INK4a (191.4%, [65.9, 116.8]) expression. Furthermore, TRPV4 knockdown increased these apoptotic and senescent markers under proinflammatory conditions compared with TRPV4 siRNA‐treated IL‐1β‐unstimulated cells (PARP, 33.1% vs. 92.9%, [−73.3, −46.4]; cleaved PARP, 289.5% vs. 119.2%, [109.9, 230.7]; cleaved caspase‐9, 199.7% vs. 164.0%, [3.69, 67.7]; p53, 279.4% vs. 127.5%, [116.9, 186.9]; p21/CIP1, 171.2% vs. 106.3%, [49.9, 72.4]; p16/INK4a, 225.8% vs. 143.1%, [78.1, 135.5]) and control siRNA‐treated IL‐1β‐stimulated cells (PARP, 33.1% vs. 70.5%, [−50.8, −24.0]; cleaved PARP, 289.5% vs. 185.1%, [44.0, 164.8]; cleaved caspase‐9, 199.7% vs. 167.0%, [0.749, 64.7]; p53, 279.4% vs. 202.5%, [41.9, 111.9]; p21/CIP1, 171.2% vs. 138.2%, [18.0, 40.5]; p16/INK4a, 225.8% vs. 191.4%, [29.8, 87.2]) (Figure 4A). These results suggest accelerated apoptosis and senescence by TRPV4 knockdown under stressful serum deprivation and inflammation.

In vitro TRPV4‐RNAi effects on disc cellular apoptosis and senescence were further evaluated by TUNEL and SA‐β‐gal staining. The percentage of TUNEL‐positive cells increased following IL‐1β stimulation (33.0% vs. 15.0%, [5.46, 30.5]), further amplified by TRPV4 RNAi (45.6% vs. 33.0%, [0.264, 25.3]) (Figure 4B). Similarly, the percentage of SA‐β‐gal‐positive cells increased following IL‐1β stimulation (38.0% vs. 21.8%, [5.98, 26.4]), amplified by TRPV4 RNAi (62.8% vs. 38.0%, [14.6, 35.0]) (Figure 4C).

### 3.4 In Vivo TRPV4 Knockdown Facilitates Prolonged Suppression of Autophagy in Rat Disc NP Tissues

Based on the in vitro cytotoxic effects of TRPV4 knockdown, in vivo intradiscal gene‐silencing experiments with TRPV4 siRNA were designed. All rats underwent surgery well and gained body weight throughout the experiment period (mean 584.7 g, [577.6, 591.9] at 56 days). All springs maintained their compressive length and fully recovered after release, indicating sustained axial loading. There found no surgery‐related complications.

**FIGURE 5:**
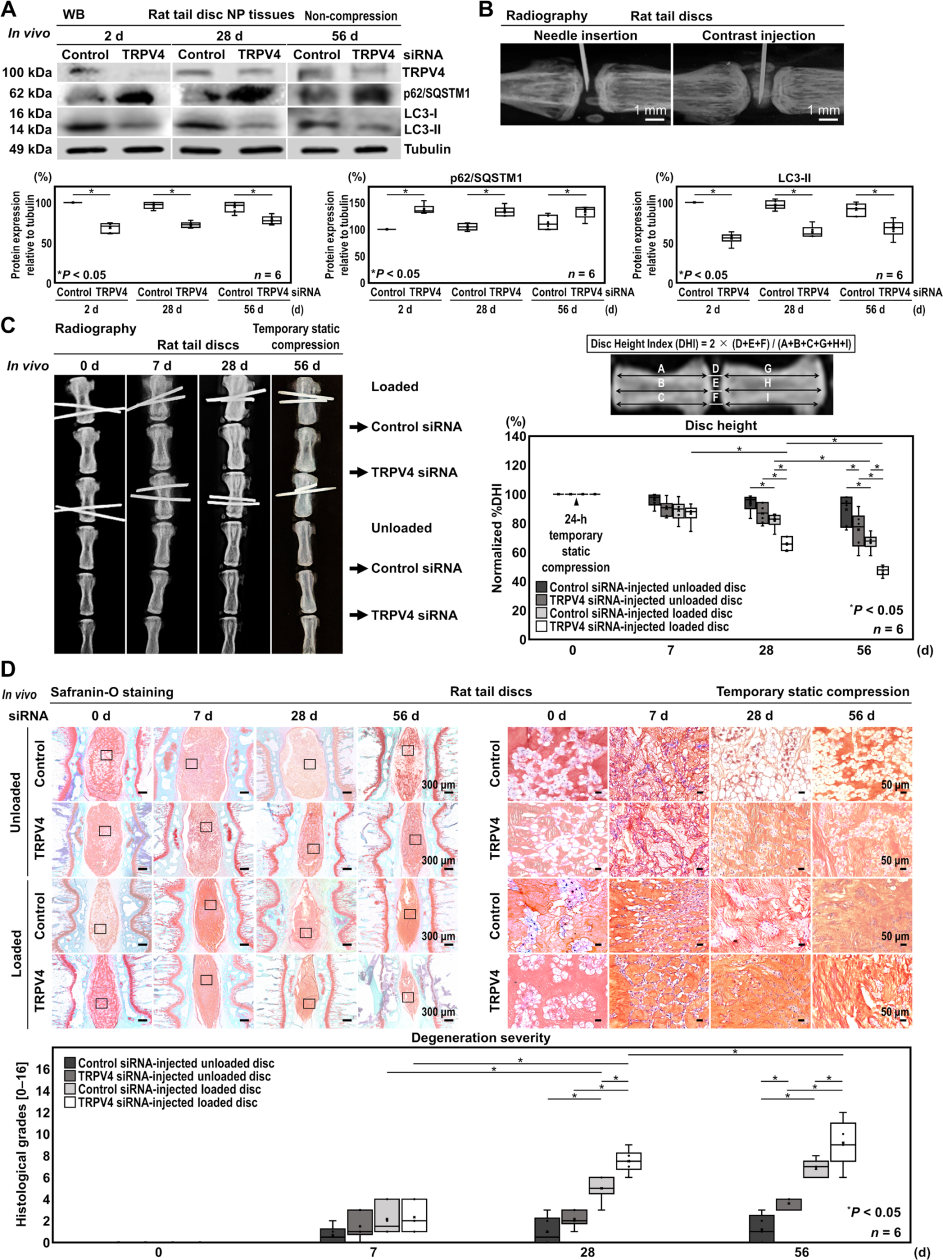
In vivo TRPV4 knockdown facilitates prolonged suppression of autophagy in rat disc NP tissues and accelerates radiographic and histomorphological disc disruption in the rat tail temporary static compression model. (A) WB for TRPV4, p62/SQSTM1, LC3‐II, and loading control tubulin of total protein extracts from rat tail disc NP tissues at 2, 28, and 56 days after TRPV4 or control siRNA injection. Changes in relative protein expression of TRPV4, p62/SQSTM1, and LC3‐II normalized to tubulin are shown. (B) Confirmation of successful intradiscal 33‐gauge needle insertion and 2‐ml contrast injection under fluoroscopic guidance. (C) Lateral radiographs of rat tail C11–12 control siRNA‐injected unloaded, C12–13 TRPV4 siRNA‐injected unloaded, C8–9 control siRNA‐injected loaded, and C9–10 TRPV4 siRNA‐injected loaded discs at 0, 7, 28, and 56 days under temporary static compression at 1.3 MPa for 24 h. Changes in the height of control siRNA‐injected unloaded, TRPV4 siRNA‐injected unloaded, control siRNA‐injected loaded, and TRPV4 siRNA‐injected loaded discs are shown. (D) Safranin‐O staining of rat tail C11–12 control siRNA‐injected unloaded, C12–13 TRPV4 siRNA‐injected unloaded, C8–9 control siRNA‐injected loaded, and C9–10 TRPV4 siRNA‐ injected loaded discs at 0, 7, 28, and 56 days under temporary static compression at 1.3 MPa for 24 h. Changes in the histopathological grade of control siRNA‐injected unloaded, TRPV4 siRNA‐injected unloaded, control siRNA‐injected loaded, and TRPV4 siRNA‐injected loaded discs are shown. In (A, C, D), data are presented with box plots (*n* = 6). In (A, C, D), two‐way ANOVA with the Tukey–Kramer post hoc test were used. Immunoblots, radiographs, and histomorphological images shown are representative of experiments with similar results.

The WB demonstrated maintained TRPV4 protein downregulation in TRPV4 siRNA‐injected discs (2 days, 31.1% knockdown [−39.2, −22.9]; 28 days, 23.9% knockdown [−32.1, −15.9]; 56 days, 16.4% knockdown [−24.5, −8.32]). Furthermore, TRPV4 RNAi showed prolonged autophagy suppression with increased p62/SQSTM1 (2 days, 137.1% [21.3, 52.8]; 28 days, 128.4% [12.7, 44.2]; 56 days, 120.2% [4.41, 35.9]) and decreased LC3‐II (2 days, 55.3% [−56.7, −32.7]; 28 days, 66.4% [−45.6, −21.6]; 56 days, 76.6% [−35.4, −11.4]) (Figure 5A). Successful administration was confirmed by radiography upon injecting a contrast agent (Figure 5B). These findings indicate extended intradiscal autophagy suppression after TRPV4 knockdown.

### 3.5 In Vivo TRPV4 Knockdown Accelerates Radiographic and Histomorphological Disc Disruption in the Rat Tail Temporary Static Compression Model

In vivo TRPV4‐RNAi effects on disc tissue disruption were evaluated under temporary static compression. In TRPV4 siRNA‐injected loaded discs, %DHI was significantly lower than TRPV4 siRNA‐injected unloaded discs at 28 days (65.8% vs. 86.8%, [−33.0, −8.99]) and 56 days (47.4% vs. 75.6%, [−40.2, −16.2]) and control siRNA‐injected loaded discs at 28 days (65.8% vs. 81.8%, [−28.0, −3.99]) and 56 days (47.4% vs. 67.1%, [−31.7, −7.75]). Meanwhile, in TRPV4 siRNA‐injected unloaded discs, %DHI was significantly lower than control siRNA‐injected unloaded discs only at 56 days (75.6% vs. 89.4%, [−25.8, −1.83]) (Figure 5C). These results displayed progressive radiological disc degeneration induced by TRPV4 RNAi with mechanical loading, suggesting involvement of TRPV4 in maintaining disc height against mechanical stress.

Similarly, safranin‐O staining presented significantly higher degenerative scores in TRPV4 siRNA‐injected loaded discs than TRPV4 siRNA‐injected unloaded discs at 28 days (7.50 vs. 2.17, [3.13, 7.54]) and 56 days (9.50 vs. 2.67, [4.63, 9.04]) and control siRNA‐injected loaded discs at 28 days (7.50 vs. 5.33, [3.13, 7.54]) and 56 days (9.50 vs. 7.00, [0.300, 4.70]). While TRPV4 siRNA‐injected unloaded discs exhibited no significant histomorphological changes compared to control siRNA‐injected unloaded discs at 7 days (1.50 vs. 0.67, [−5.07, 17.2]) and 28 days (2.17 vs. 1.00, [−3.89, 18.3]). However, TRPV4 siRNA‐injected unloaded discs exhibited significant histomorphological deterioration compared to control siRNA‐injected unloaded discs at 56 days (2.67 vs. 1.17, [−4.60, −0.07]) (Figure 5D). These results implied that under loading conditions TRPV4 RNAi induced intervertebral disc degeneration more rapidly and severely over 56 days compared to TRPV4 suppression without loading or loading alone.

### 3.6 In Vivo TRPV4 Knockdown Suppresses ECM Metabolism, AMPK Phosphorylation, Autophagy Activity in the Rat Tail Temporary Static Compression Model

**FIGURE 6:**
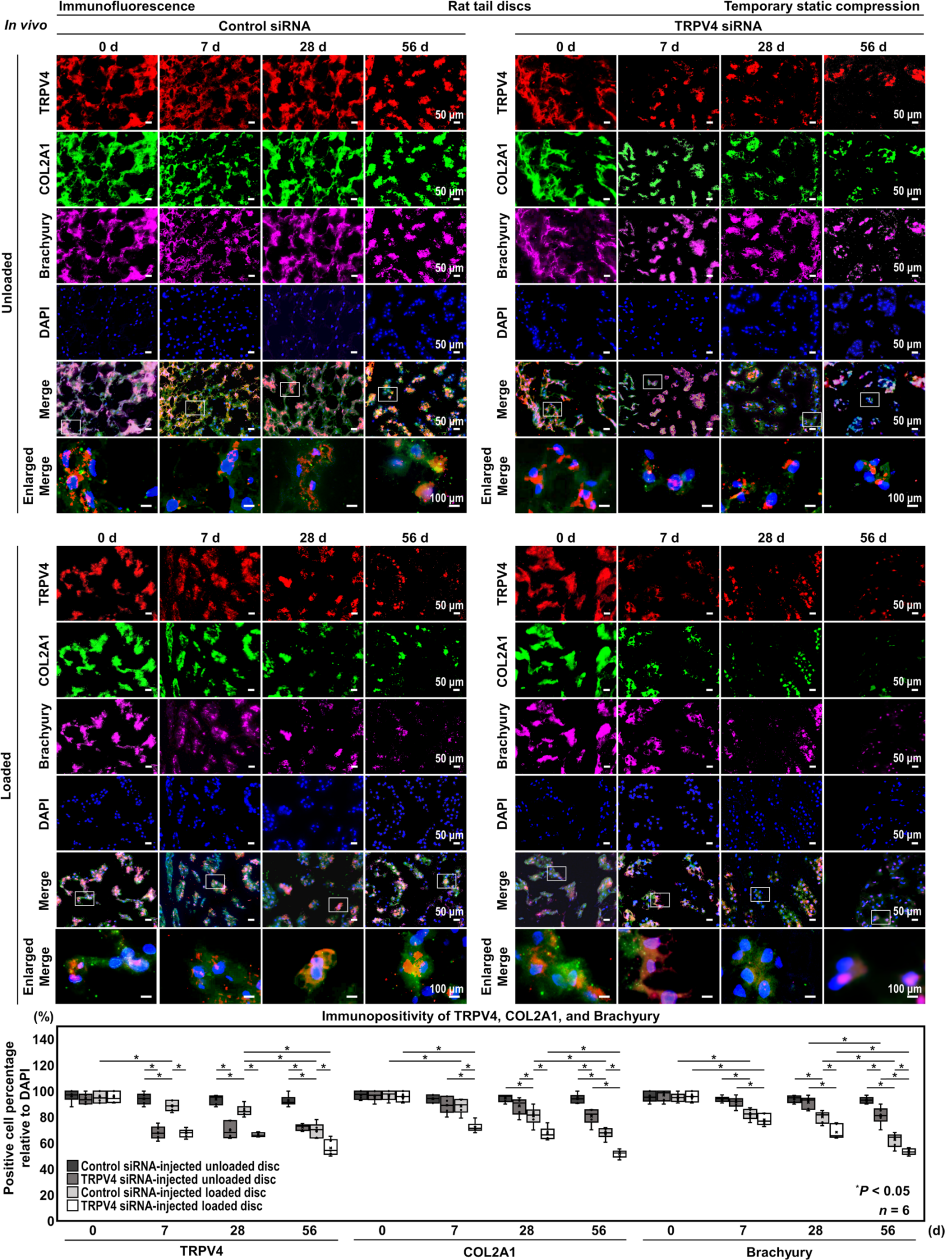
In vivo TRPV4 knockdown suppresses ECM metabolism in the rat tail temporary static compression model. Immunofluorescence for TRPV4 (red), COL2A1 (green), phenotypic Brachyury (purple), nuclear DAPI (blue), and merged signals of rat tail C11–12 control siRNA‐injected unloaded, C12–13 TRPV4 siRNA‐injected unloaded, C8–9 control siRNA‐injected loaded, and C9–10 TRPV4 siRNA‐injected loaded discs at 0, 7, 28, and 56 days under temporary static compression at 1.3 MPa for 24 h. Changes in the percentage of TRPV4‐positive, COL2A1‐positive, and Brachyury‐positive cells relative to DAPI‐positive cells of control siRNA‐injected unloaded, TRPV4 siRNA‐injected unloaded, control siRNA‐injected loaded, and TRPV4 siRNA‐injected loaded discs are shown. Cell count was performed in respective four random LPFs of duplicates. Data are presented with box plots (*n* = 6). Two‐way ANOVA with the Tukey–Kramer post hoc test was used. Immunofluorescent images shown are representative of experiments with similar results.

We finally performed multicolor immunofluorescence for TRPV4, ECM metabolism, AMPK phosphorylation, and autophagy activity in the rat tail temporary static compression model. Immunopositivity for TRPV4 was significantly lower in TRPV4 siRNA‐injected discs than in control siRNA‐injected ones under unloaded (7 days, 68.0% vs. 94.1%, [−34.9, −17.3]; 28 days, 66.5% vs. 88.7%, [−32.6, −15.0]; 56 days, 72.2% vs. 92.5%, [−29.0, −11.4]) and loaded conditions (7 days, 67.5% vs. 88.7%, [−29.9, −12.3]; 28 days, 66.5% vs. 84.9%, [−27.3, −9.66]; 56 days, 56.2% vs. 69.9%, [−22.4, −4.82]). In TRPV4 siRNA‐injected loaded discs, the percentage of COL2A1‐positive cells was significantly lower than in TRPV4 siRNA‐injected unloaded discs at all time points (7 days, 72.2% vs. 89.2%, [−26.4, −7.55]; 28 days, 67.3% vs. 88.6%, [−30.7, −11.9]; 56 days, 51.7% vs. 80.7%, [−38.4, −19.6]) and in control siRNA‐injected loaded discs at all time points (7 days, 72.2% vs. 88.5%, [−25.6, −6.83]; 28 days, 67.3% vs. 80.9%, [−23.0, −4.22]; 56 days, 51.7% vs. 67.5% [−25.2, −6.41]). The percentage of Brachyury‐positive cells in loaded discs was lower than that in unloaded discs at all time points (control siRNA: 7 days, 82.2% vs. 93.9%, [−21.9, −4.93]; 28 days, 80.1% vs. 94.0%, [−24.1, −7.10]; 56 days, 62.6% vs. 93.0%, [−41.6, −24.6], TRPV4 siRNA: 7 days, 77.9% vs. 91.6%, [−22.2, −5.19]; 28 days, 68.4% vs. 91.3%, [−31.5, −14.5]; 56 days, 53.3% vs. 81.2%, [−36.3, −19.3]). In TRPV4 siRNA‐injected unloaded discs, the percentage of COL2A1‐positive cells did not significantly decrease compared with control siRNA‐injected unloaded discs at 7 days (89.2% vs. 94.1%, [−14.3, 4.47]) and 28 days (88.6% vs. 94.7%, [−15.5, 3.27]), however, significantly decreased at 56 days (80.7% vs. 94.2%, [−22.9, −4.09]) with a decrease in Brachyury expression (81.2% vs. 93.0%, [−20.4, −3.41]) (Figure 6).

**FIGURE 7:**
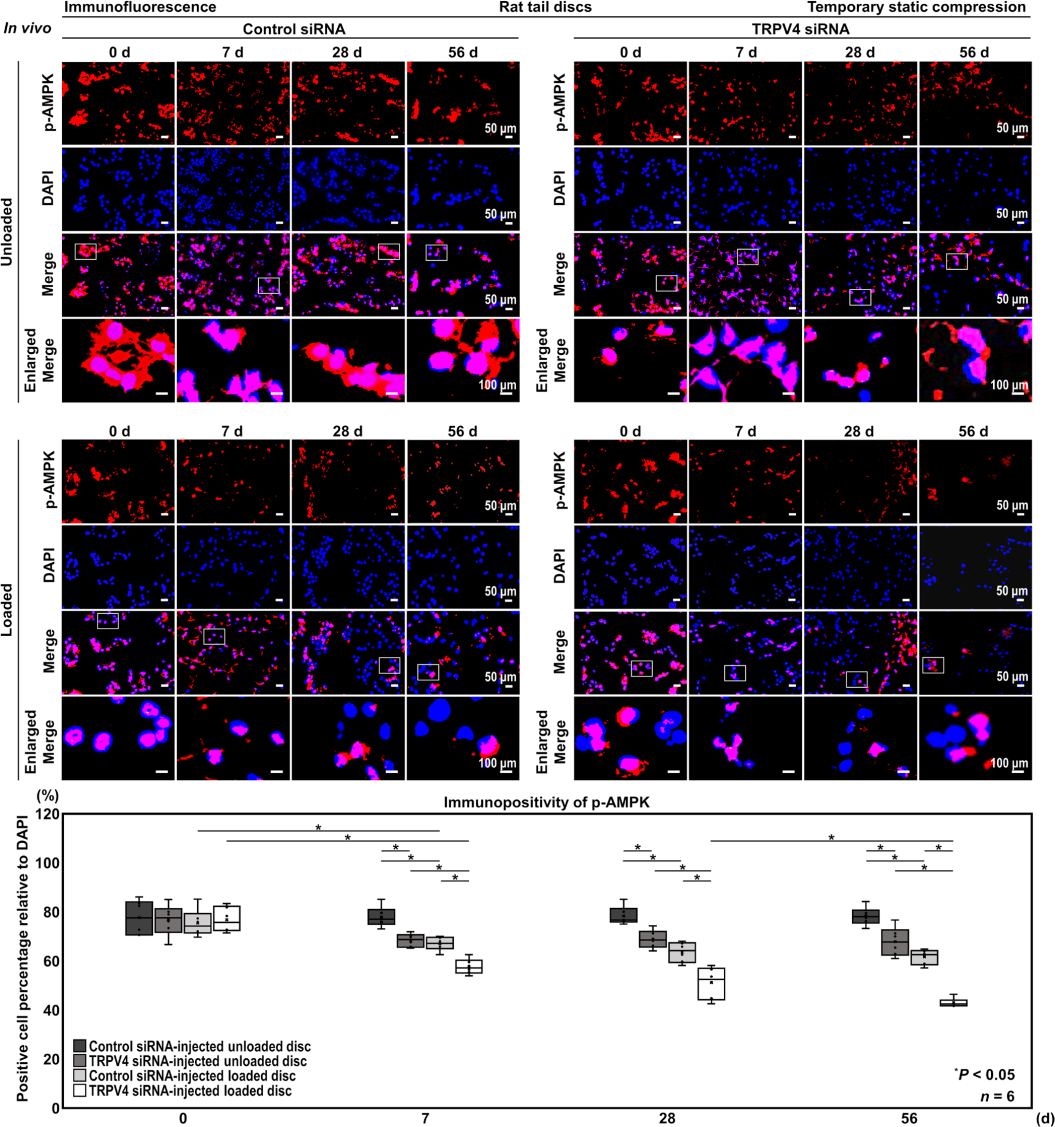
In vivo TRPV4 knockdown suppresses AMPK phosphorylation in the rat tail temporary static compression model. Immunofluorescence for p‐AMPK (red) and nuclear DAPI (blue), and merged signals of rat tail C11–12 control siRNA‐injected unloaded, C12–13 TRPV4 siRNA‐injected unloaded, C8–9 control siRNA‐injected loaded, and C9–10 TRPV4 siRNA‐injected loaded discs at 0, 7, 28, and 56 days under temporary static compression at 1.3 MPa for 24 h. Changes in the percentage of p‐AMPK‐positive cells relative to DAPI‐positive cells of control siRNA‐injected unloaded, TRPV4 siRNA‐injected unloaded, control siRNA‐injected loaded, and TRPV4 siRNA‐injected loaded discs are shown. Cell count was performed in respective four random LPFs of duplicates. Data are presented with box plots (*n* = 6). Two‐way ANOVA with the Tukey–Kramer post hoc test was used. Immunofluorescent images shown are representative of experiments with similar results.

In TRPV4 siRNA‐injected unloaded discs, the immunopositivity of p‐AMPK significantly decreased compared with control siRNA‐injected unloaded discs at all time points (7 days, 68.4% vs. 77.8%, [−18.7, −0.157]; 28 days, 68.8% vs. 78.3%, [−18.6, 0.161]; 56 days, 67.8% vs. 78.2% [−19.6, −1.04]), and in TRPV4 siRNA‐injected loaded discs, the percentage of p‐AMPK‐positive cells was significantly lower than in TRPV4 siRNA‐injected unloaded discs at all time points (7 days, 60.3% vs. 72.1%, [−21.0, −2.63]; 28 days, 55.5% vs. 77.0%, [−23.8, −5.39]; 56 days, 43.0% vs. 60.6%, [−26.8, −8.38]) and in control siRNA‐injected loaded discs at all time points (7 days, 57.6% vs. 67.0%, [−18.7, −0.092]; 28 days, 51.1% vs. 63.6%, [−21.7, −3.13]; 56 days, 43.0% vs. 61.6%, [−27.9, −9.35]) (Figure 7).

**FIGURE 8:**
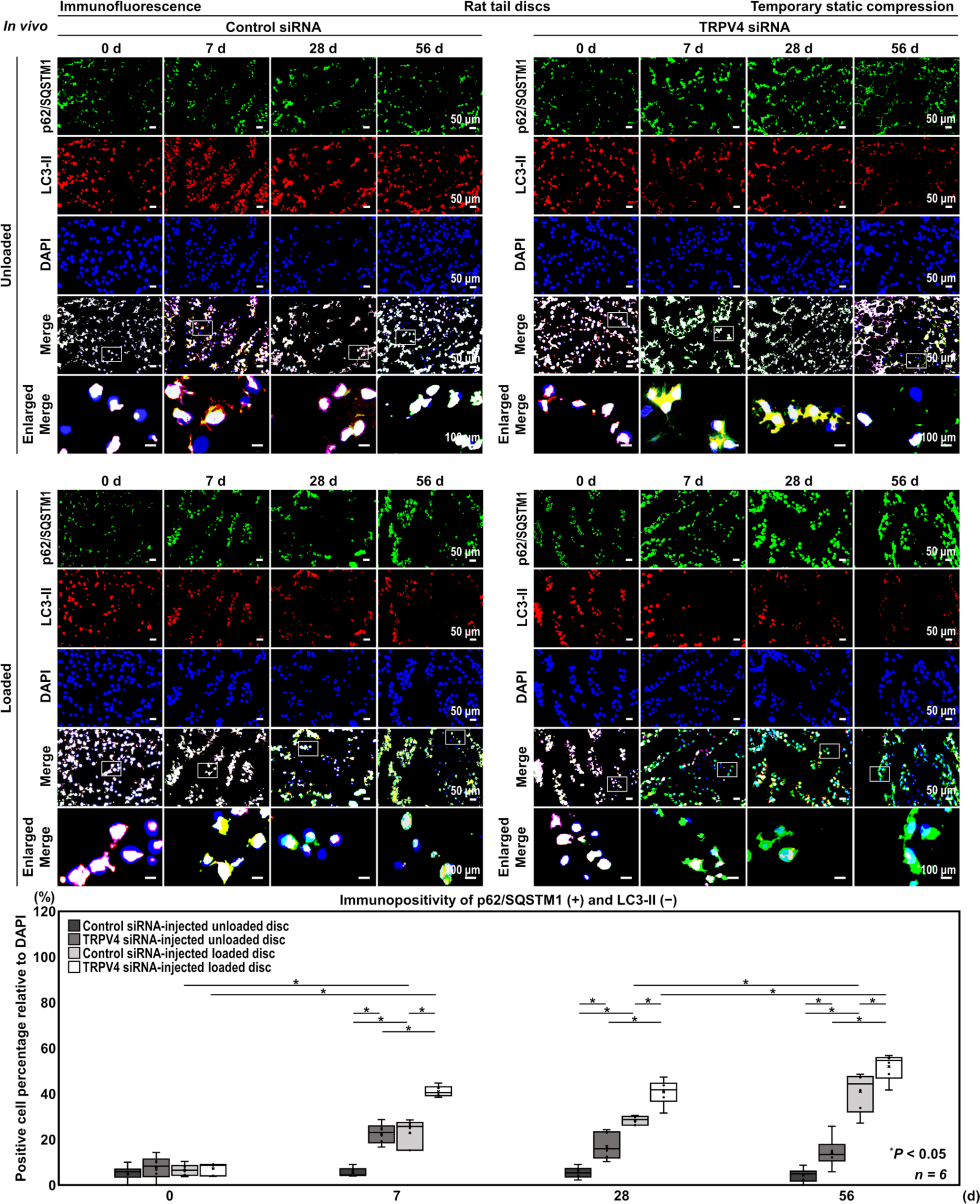
In vivo TRPV4 knockdown suppresses autophagy activity in the rat tail temporary static compression model. Immunofluorescence for p62/SQSTM1 (green), LC3‐II (red), nuclear DAPI (blue), and merged signals of rat tail C11–12 control siRNA‐injected unloaded, C12–13 TRPV4 siRNA‐injected unloaded, C8–9 control siRNA‐injected loaded, and C9–10 TRPV4 siRNA‐injected loaded discs at 0, 7, 28, and 56 days under temporary static compression at 1.3 MPa for 24 h. Changes in the percentage of p62/SQSTM1‐positive, LC3‐II‐positive, and p62/SQSTM1‐positive and LC3‐II‐negative cells relative to DAPI‐positive cells of control siRNA‐injected unloaded, TRPV4 siRNA‐injected unloaded, control siRNA‐injected loaded, and TRPV4 siRNA‐injected loaded discs are shown. Cell count was performed in respective four random LPFs of duplicates. Data are presented with box plots (*n* = 6). Two‐way ANOVA with the Tukey–Kramer post hoc test was used. Immunofluorescent images shown are representative of experiments with similar results.

In addition, in TRPV4 siRNA‐injected unloaded discs, the percentage of p62/SQSTM1‐positive and LC3‐II‐negative cells significantly decreased compared with control siRNA‐injected unloaded discs (7 days, 22.6% vs. 5.46%, [7.74, 26.6]; 28 days, 17.0% vs. 5.41%, [2.08, 21.0]; 56 days, 14.3% vs. 4.38%, 0.469% vs. 19.3% [21.9, 42.1]), and in TRPV4 siRNA‐injected loaded discs, the percentage of p62/SQSTM1‐positive and LC3‐II‐negative cells was significantly higher than in TRPV4 siRNA‐injected unloaded discs (7 days, 41.0% vs. 22.6%, [8.95, 27.8]; 28 days, 40.8% vs. 17.0%, [184.4, 33.2]; 56 days, 52.0% vs. 14.3% [28.2, 47.1]) and in control siRNA‐injected loaded discs (7 days, 41.0% vs. 22.9%, [8.68, 27.6]; 28 days 40.8% vs. 28.4%, [2.91, 21.8]; 56 days, 52.0% vs. 40.9%, [1.61, 20.5]) (Figure 8).

Collectively, the observed findings support the TRPV4 siRNA‐mediated deceleration of ECM metabolism via autophagy suppression in the intervertebral disc homeostasis, particularly under mechanical stress.

## 4 Discussion

This is the first study showing in vitro and in vivo TRPV4 RNAi‐mediated autophagy and ECM metabolism suppression, resulting in in vivo progressive disc disruptive changes. In this study, we applied proinflammatory stimulation to disc NP cells in vitro and used a rat compression model in vivo to conduct stress‐loaded experiments [7, 18].

In vitro, first, we confirmed the autophagy inhibition through suppression of the AMPK/mTOR pathway by TRPV4 knockdown using WB. We also found that TRPV4 knockdown suppressed ECM metabolism under proinflammatory stimulation. Second, TRPV4 knockdown significantly increased apoptosis and senescence under proinflammatory stimulation, resulting in approximately 40% cell viability. This is consistent with the previous studies, showing that Atg5 inhibition promotes apoptosis and senescence activity [15]. Additionally, the cell viability markedly decreased both under serum deprivation and proinflammatory stimulation by TRPV4 RNAi. This in vitro study indicates anabolic, antiapoptotic, and antisenescent effects of TRPV4 in the intradiscal homeostasis via autophagy.

In vivo, the knockdown effect by TRPV4 siRNA was maintained in the disc NP tissue through 56 days after injection. The reported siRNA‐retaining period in rat disc NP cells and tissues varies from 2–3 [32] to 8 weeks [15]. This notable feature could be due to the anatomical and biological characteristics of the disc [32]. Our in vivo results were consistent with in vitro results; the observed mechanically induced radiographic, histomorphological, and immunofluorescent disc disruption was further accelerated by TRPV4 knockdown with autophagy suppression through AMPK downregulation. The lowest disc height, highest degeneration grade, and downregulation of AMPK, autophagy, and COL2A1 were observed in TRPV4 siRNA‐injected loaded discs. Previously, the relationship between mechanical stress and TRPV4 [16] and the involvement of autophagy in ECM metabolism [28] have been investigated, respectively. However, we examined the connections among these four elements and suggested that TRPV4 contributes to maintaining intradiscal homeostasis against physical stresses via autophagy.

This study has several limitations. First, our rat tail model does not exactly mimic human aging, and rats retain disc notochordal cells throughout their lives unlike humans. Second, we used siRNA to suppress TRPV4. While siRNA exhibited stable and enough knockdown, we cannot control its knockdown effect. Metabolic effect by TRPV4 knockdown could vary according to the extent of the knockdown [19, 46]. TRPV4 is activated by hydrostatic pressure within physiological ranges in vitro [16, 17]; however, in the present study, TRPV4 activity decreased under the intense load of 1.3 MPa in vivo. Third, the expression profile of TRPV4 in degenerating human intervertebral discs remains unclear. We have not yet demonstrated how TRPV4 works during tissue degeneration. Moderate TRPV4 activation may be beneficial for maintaining disc homeostasis, while excessive or dysregulated TRPV4 signaling can contribute to inflammation and degeneration [47]. We are now conducting a gain‐of‐function study on TRPV4. This study will strongly support that TRPV4 modulation has a potential to be a therapeutic target.

In conclusion, the in vitro and in vivo loss‐of‐function study of TRPV4 in the rat intervertebral disc suggests a possible therapeutic strategy by modulating TRPV4 activity in the disc degenerative diseases.

## Author Contributions

T.M. and Y.T.: conception/design of the work, acquisition/analysis/interpretation of data, drafting of the manuscript, final approval of the manuscript, agreement with accuracy/integrity. T.T., T.Y., K.M., H.O., M.R., N.K., K.K., and Y.H.: analysis/interpretation of data, critical revising of the manuscript, final approval of the manuscript, agreement with accuracy/integrity. R.K. and K.K.: conception/design of the work, analysis/interpretation of data, drafting/critical revising of the manuscript, final approval of the manuscript, agreement with accuracy/integrity.

## Conflicts of Interest

Y.T. and K.K. are the faculty members of the endowed course by Surgical Spine Inc. and SMI Japan Inc.

## Supporting information


Data S1.

Data S2.
