# Supplementary material for: Transient Receptor Potential Vanilloid 4 Knockdown Decreases Extracellular Matrix Synthesis via Autophagy Suppression in the Rat Intervertebral Disc
Source: JOR Spine. 2025 Feb 17;8(1):e70046. doi: 10.1002/jsp2.70046 (PMC11832302; doi:10.1002/jsp2.70046)
Supplement: Supplementary file 1 — Data S1. [file JSP2-8-e70046-s002.pdf]

**Supplemental Table 1.** List of the antibodies, reagents, and instruments used.

| Product | | Catalog number | Manufacturer |
|---|---|---|---|
| *Antibody* | | | |
| TRPV1 (rabbit, polyclonal) | | ACC-030 | Almone labs (Jerusalem, Israel) |
| TRPV4 (rabbit, polyclonal) | | ACC-124 | Almone labs (Jerusalem, Israel) |
| TRPV5 (rabbit, polyclonal) | | ACC-035 | Almone labs (Jerusalem, Israel) |
| AMPK (rabbit, polyclonal) | | 2532S | Cell Signaling Technology (Danvers, MA) |
| P-AMPK (rabbit, monoclonal) | | 2535S | Cell Signaling Technology (Danvers, MA) |
| mTOR (rabbit, monoclonal) | | 2983 | Cell Signaling Technology (Danvers, MA) |
| RAPTOR (rabbit, monoclonal) | | 2280 | Cell Signaling Technology (Danvers, MA) |
| RICTOR (rabbit, monoclonal) | | 2114 | Cell Signaling Technology (Danvers, MA) |
| p70/S6K (rabbit, monoclonal) | | 2708 | Cell Signaling Technology (Danvers, MA) |
| LC3 (rabbit, monoclonal) | | 3868 | Cell Signaling Technology (Danvers, MA) |
| p62/SQSTM1 (mouse, monoclonal) | | ab56416 | Abcam (Cambridge, UK) |
| Cleaved caspase-9 (rabbit, polyclonal) | | 9507 | Cell Signaling Technology (Danvers, MA) |
| PARP (rabbit, monoclonal) | | 9532 | Cell Signaling Technology (Danvers, MA) |
| Cleaved PARP (rabbit, monoclonal) | | 5625 | Cell Signaling Technology (Danvers, MA) |
| p53 (mouse, monoclonal) | | 2524 | Cell Signaling Technology (Danvers, MA) |
| p21/CIP1 (mouse, monoclonal) | | sc-6246 | Santa Cruz Biotechnology (Santa Cruz, CA) |
| Brachyury (mouse, monoclonal) | | sc-166962 | Santa Cruz Biotechnology (Santa Cruz, CA) |
| CD24 (mouse, monoclonal) | | sc-19585 | Santa Cruz Biotechnology (Santa Cruz, CA) |
| p16/INK4a (mouse, monoclonal) | | sc-1661 | Santa Cruz Biotechnology (Santa Cruz, CA) |
| COL2A1 (mouse, monoclonal) | | sc-52658 | Santa Cruz Biotechnology (Santa Cruz, CA) |
| Aggrecan (mouse, monoclonal) | | ab3778 | Abcam (Cambridge, UK) |
| MMP3 (rabbit, monoclonal) | | ab52915 | Abcam (Cambridge, UK) |
| MMP13 (rabbit, polyclonal) | | ab39012 | Abcam (Cambridge, UK) |
| TIMP1 (mouse, monoclonal) | | sc-21734 | Santa Cruz Biotechnology (Santa Cruz, CA) |
| TIMP2 (rabbit, monoclonal) | | 5738 | Cell Signaling Technology (Danvers, MA) |
| Tubulin (mouse, monoclonal) | | T9026 | Sigma-Aldrich (St. Louis, MO) |
| *RNAi* | | | |
| Lipofectamine RNAiMAX transfection reagent | | 13778150 | Thermo Fisher Scientific (Waltham, MA) |
| Invivofectamine™ 3.0 Reagent | | IVF3001 | Thermo Fisher Scientific (Waltham, MA) |
| Opti-minimal essential medium I | | 3198070 | Thermo Fisher Scientific (Waltham, MA) |
| Stealth RNAi siRNA, negative control | | 10620312-367123 D11/E02 | Thermo Fisher Scientific (Waltham, MA) |
| Stealth RNAi siRNAs, TRPV4 | Sequence 1 | RSS330372 | Thermo Fisher Scientific (Waltham, MA) |
| | Sequence 2 | RSS330373 | |
| | Sequence 3 | RSS330374 | |

| Product | Catalog number | Manufacturer |
|---|---|---|
| *Cell culture* | | |
| DMEM | D5796 | Sigma-Aldrich (St. Louis, MO) |
| FBS | F2442 | Sigma-Aldrich (St. Louis, MO) |
| Penicillin/streptomycin | 26253-84 | Nacalai Tesque (Kyoto, Japan) |
| Collagenase type 2 | LS004176 | Worthington Biochemical (Lakewood, NJ) |
| Rat recombinant IL-1β | 092-04263 | Wako (Osaka, Japan) |
| CCK-8 | CK04 | Dojindo Laboratories (Kumamoto, Japan) |
| Model 680 microplate reader | | Bio-Rad (Hercules, CA) |
| *Staining* | | |
| Hematoxylin | 30002 | Muto (Tokyo, Japan) |
| Eosin | 058-00062 | Wako (Osaka, Japan) |
| Safranin-O | S-0145 | Tokyo Chemical Industry (Tokyo, Japan) |
| Fast green | 10720 | Chroma Gesellschaft Schmidt (Munster, Germany) |
| *In situ* cell death detection kit, fluorescein | 11684795910 | Roche (Basel, Switzerland) |
| SA-β-gal staining kit | 9860 | Cell Signaling Technology (Danvers, MA) |
| 4% Paraformaldehyde phosphate buffer solution | 163-20145 | Wako (Osaka, Japan) |
| Alexa Fluor® 488 secondary antibody | A-21467 | Thermo Fisher Scientific (Waltham, MA) |
| Alexa Fluor® 568 secondary antibody | A-10042 | Thermo Fisher Scientific (Waltham, MA) |
| Alexa Fluor® 647 secondary antibody | A-31571 | Thermo Fisher Scientific (Waltham, MA) |
| Rat IgG Isotype Control | 31933 | Thermo Fisher Scientific (Waltham, MA) |
| DAPI | D1306 | Thermo Fisher Scientific (Waltham, MA) |
| BZ-X700 microscope | | Keyence (Osaka, Japan) |
| *Western blotting* | | |
| 3-(*N*-Morpholino)propanesulfonic acid | 23438-64 | Nacalai Tesque (Kyoto, Japan) |
| Protease inhibitor cocktail | 25955-11 | Nacalai Tesque (Kyoto, Japan) |
| Tris(hydroxymethyl)aminomethane | 35434-21 | Nacalai Tesque (Kyoto, Japan) |
| Chemi-Lumi One Super | 02230-30 | Nacalai Tesque (Kyoto, Japan) |
| Phosphatase inhibitor cocktails 2 | P5726 | Sigma-Aldrich (St. Louis, MO) |
| Phosphatase inhibitor cocktails 3 | P0044 | Sigma-Aldrich (St. Louis, MO) |
| Glycine | 12-1210 | Sigma-Aldrich (St. Louis, MO) |
| T-PER tissue protein extraction reagent | 78510 | Thermo Fisher Scientific (Waltham, MA) |
| Pierce BCA protein assay kit | 23227 | Thermo Fisher Scientific (Waltham, MA) |
| 7.5%–15.0% Polyacrylamide gel | SDG-581 | Bio Craft (Tokyo, Japan) |
| 4× Laemmli sample buffer | 1610747 | Bio-Rad (Hercules, CA) |
| 2-Mercaptoethanol | 21438-82 | Nacalai Tesque (Kyoto, Japan) |
| SDS | 191-07145 | Wako (Osaka, Japan) |
| 0.2 PVDF Western blotting membrane | 10600021 | GE Healthcare (Chicago, IL) |
| Anti-rabbit secondary antibody | NA934 | GE Healthcare (Chicago, IL) |

| Product | Catalog number | Manufacturer |
|---|---|---|
| *Western blotting (continued)* | | |
| Anti-mouse secondary antibody | NA931 | GE Healthcare (Chicago, IL) |
| MS-100R bead-beating disrupter | | Tomy Seiko (Tokyo, Japan) |
| LAS-3000 mini | | Fujifilm (Tokyo, Japan) |
| *Radiography* | | |
| IXFR film | | Fujifilm (Tokyo, Japan) |
| VPX-30E radiography system | | Toshiba Medical Supply (Tokyo, Japan) |

AMPK = AMP-activated protein kinase; CCK-8 = cell counting kit-8; COL2A1 = collagen type II alpha 1; DAPI = 4',6-A diamidino-2-phenylindole; DMEM = Dulbecco's modified Eagle's medium; FBS = fetal bovine serum; IL-1β = interleukin-1 beta; LC3 = light chain 3; MMP = matrix metalloproteinase; p62/SQSTM1 = p62/sequestosome 1; p70/S6K = p70/ribosomal S6 kinase; p-AMPK = phospho-AMPK; PARP = poly (ADP-ribose) polymerase; PVDF = polyvinylidene difluoride; RNAi = RNA interference; SA-β-gal = senescence associated beta galactosidase; SDS = sodium dodecyl sulfate; siRNA = small interfering RNA; TIMP = tissue inhibitors of metalloproteinase; TRPV4 = Trancient receptor potential vanilloid 4.

**Supplemental Table 2.** List of small interfering RNA sequences used.

| siRNA | | | Sequence (5' to 3') |
|---|---|---|---|
| TRPV4 | No. 1 | Sense | GCAAGAUCGAGAACCGCCAUGAGAU |
| | | Anti-sense | AUCUCAUGGCGGUUCUCGAUCUUGC |
| | No. 2 | Sense | CACCUGUCUCGCAAGUUCAAGGACU |
| | | Anti-sense | AGUCCUUGAACUUGCGAGACAGGUG |
| | No. 3 | Sense | UCCUUCCAGUUGCUCUACUUCAUCU |
| | | Anti-sense | AGAUGAAGUAGAGCAACUGGAAGGA |
| Negative control | | Sense | Same as Stealth RNAi Negative Control Med GC Duplex siRNA |
| | | Anti-sense | (12935300) and confidential by Thermo Fisher Scientific (Waltham, MA) |

All siRNAs are custom designed for *in-vitro* and *in-vivo* purity.

TRPV4 = Trancient receptor potential vanilloid 4; RNAi = RNA interference; siRNA = small interfering RNA.
