# Supplementary material for: Transient Receptor Potential Vanilloid 4 Knockdown Decreases Extracellular Matrix Synthesis via Autophagy Suppression in the Rat Intervertebral Disc
Source: JOR Spine. 2025 Feb 17;8(1):e70046. doi: 10.1002/jsp2.70046 (PMC11832302; doi:10.1002/jsp2.70046)
Supplement: Supplementary file 2 — Data S2. [file JSP2-8-e70046-s001.pdf]

Figure 1E

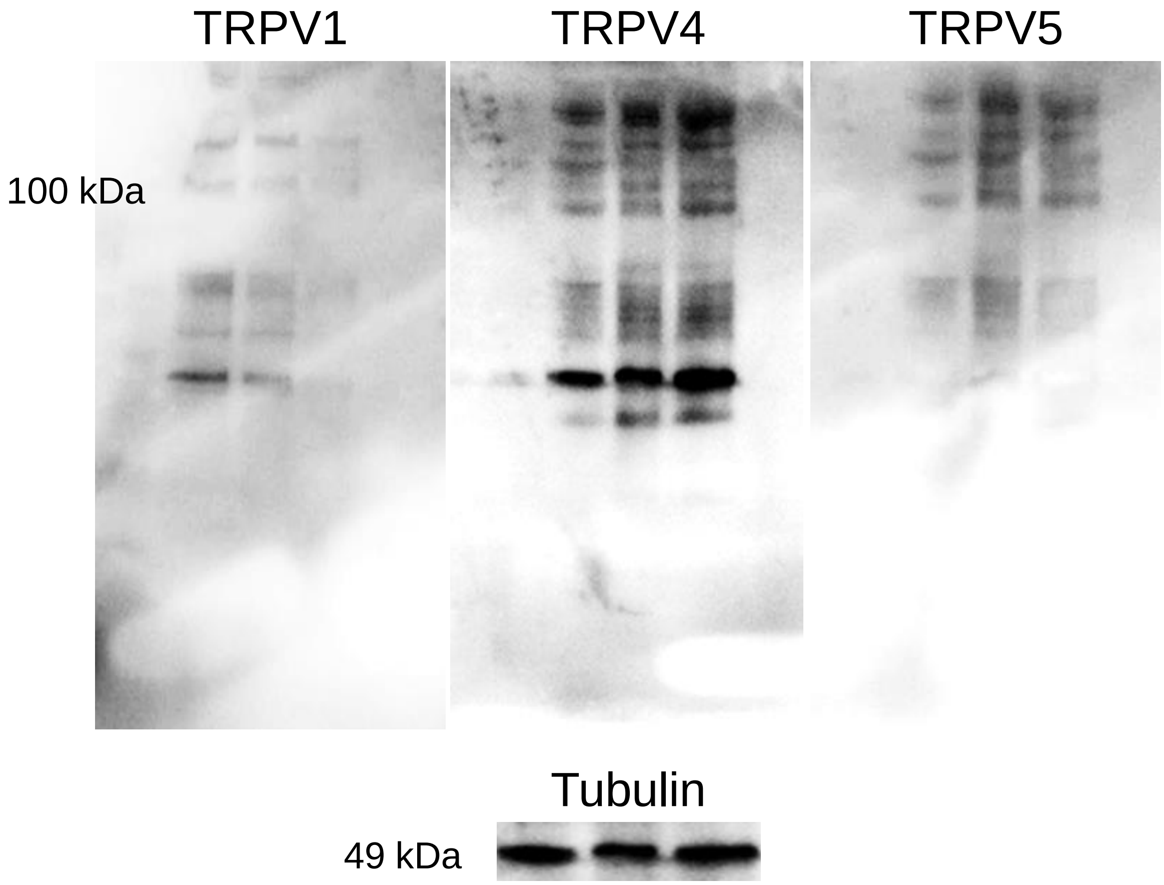

Figure 2A

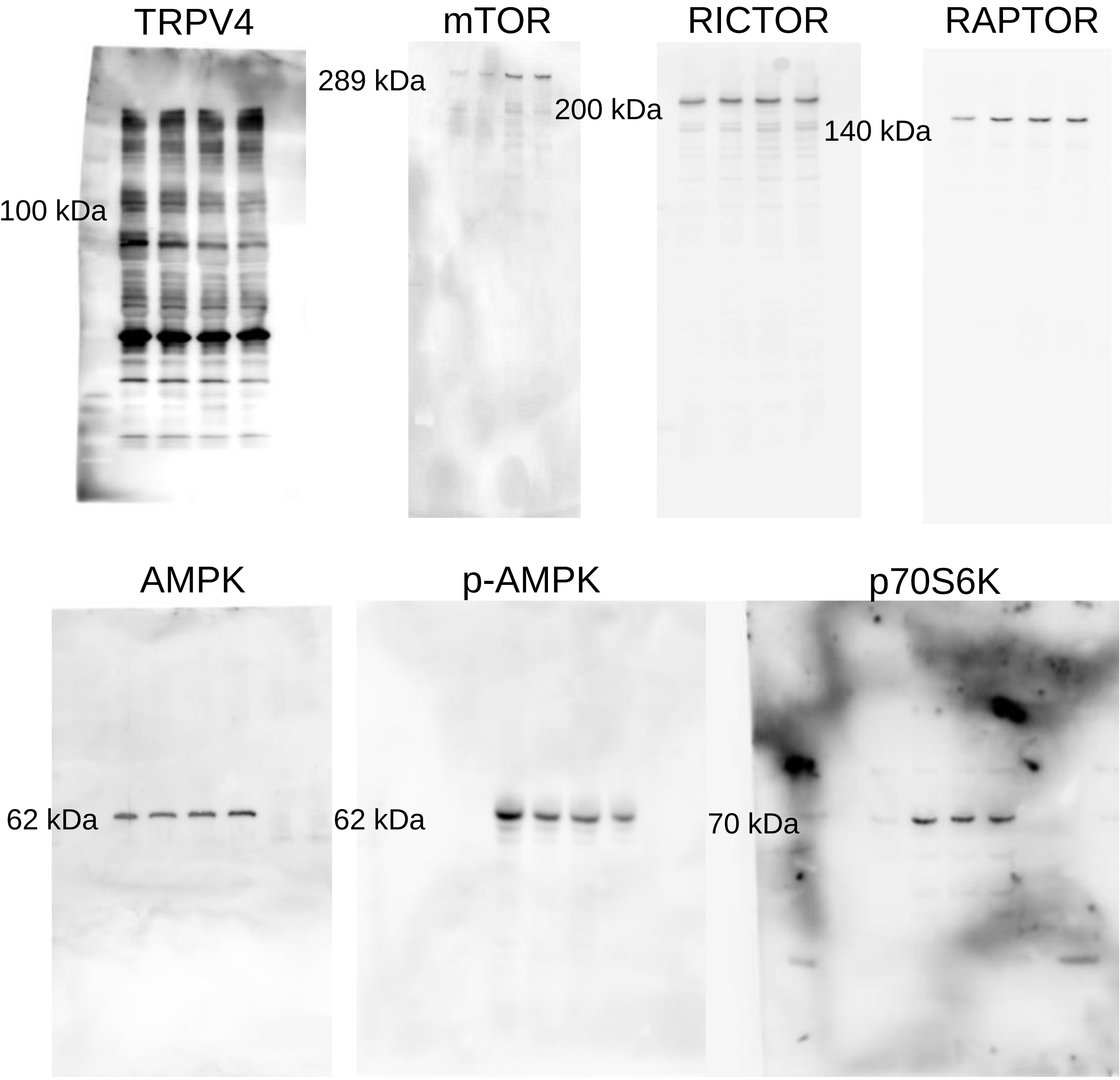

TRPV4
mTOR
RICTOR
RAPTOR
289 kDa
200 kDa
140 kDa
100 kDa
AMPK
p-AMPK
p70S6K
62 kDa
62 kDa
70 kDa

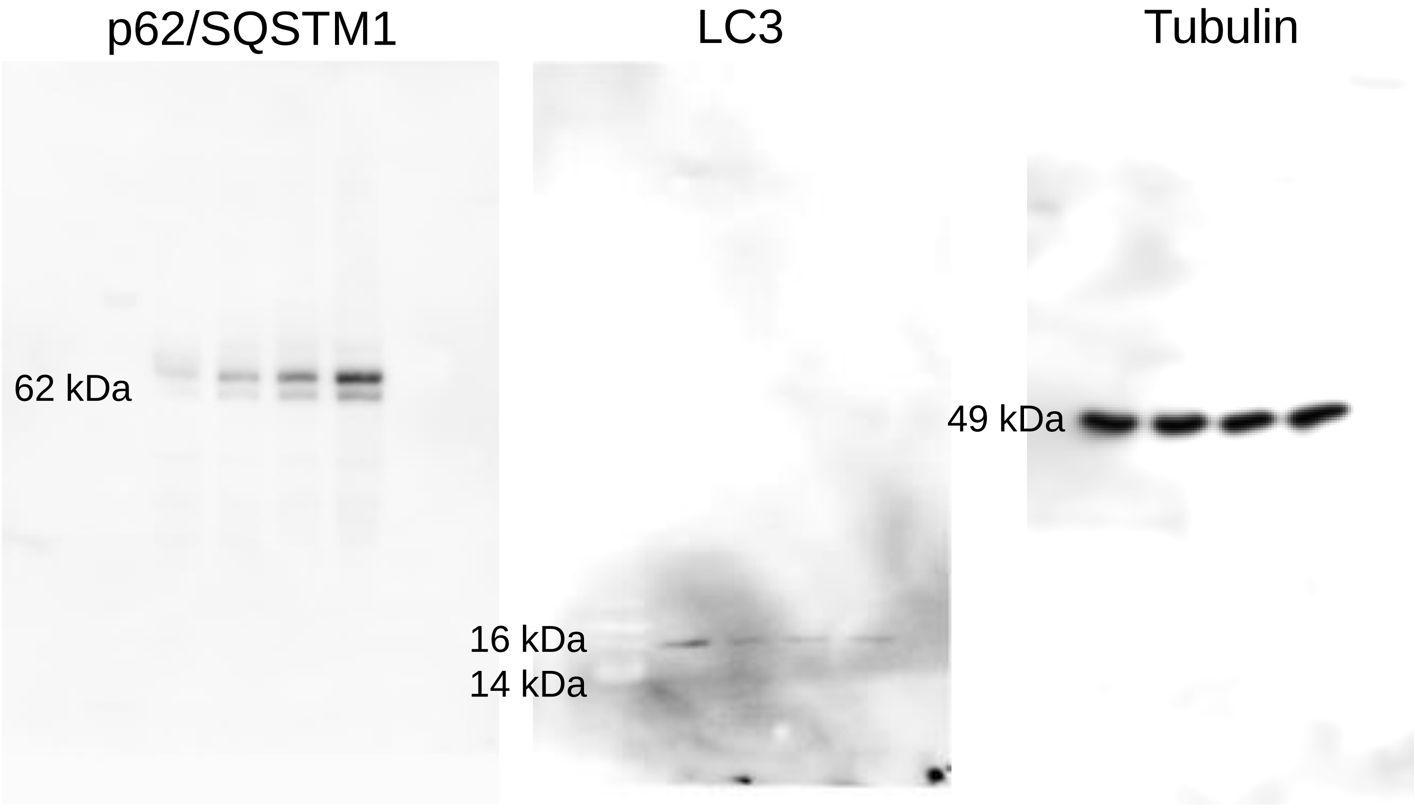
p62/SQSTM1
LC3
Tubulin
62 kDa
16 kDa
14 kDa
49 kDa

Figure 3

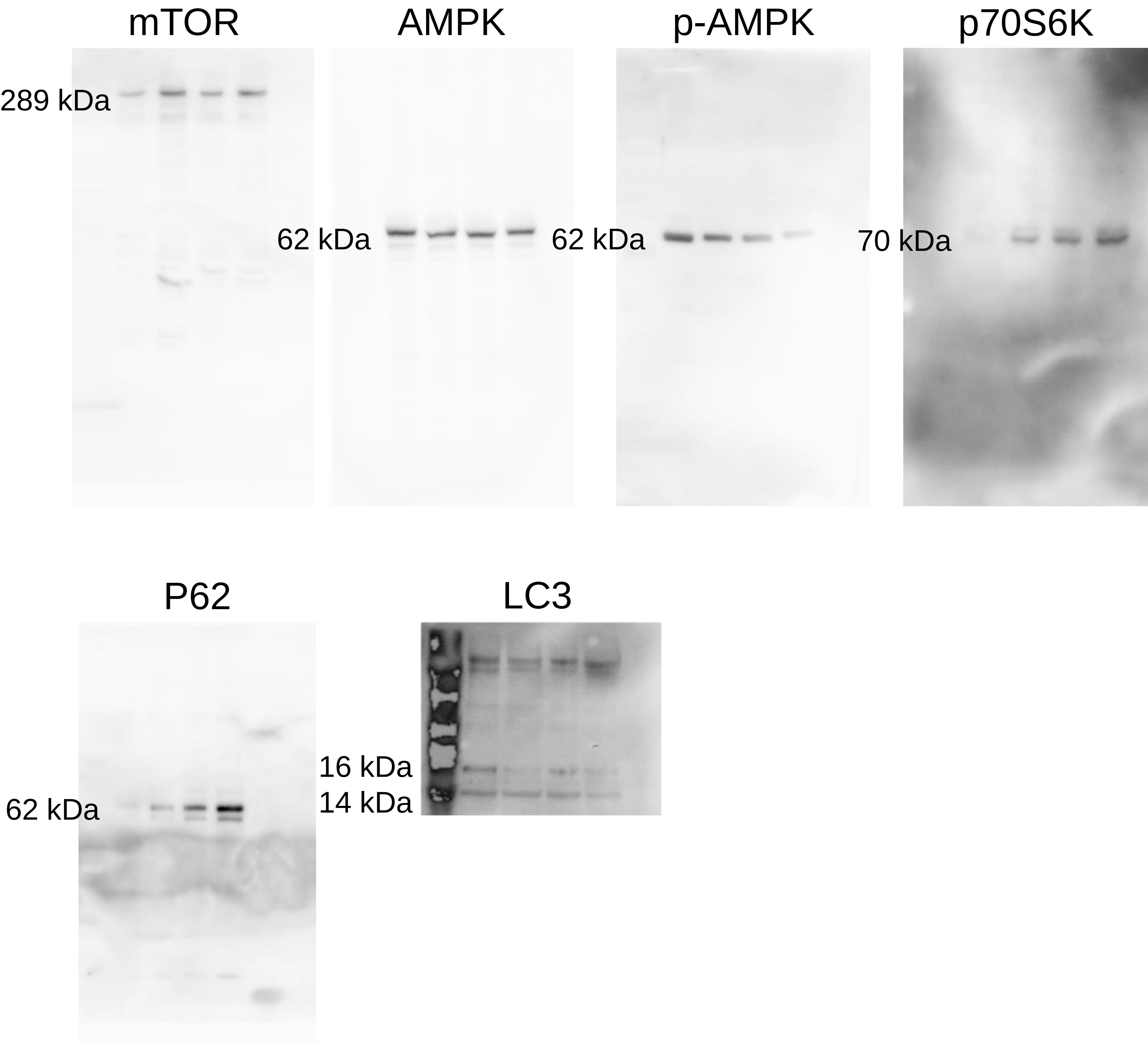

mTOR
AMPK
p-AMPK
p70S6K
289 kDa
62 kDa
62 kDa
70 kDa
P62
LC3
16 kDa
14 kDa
62 kDa

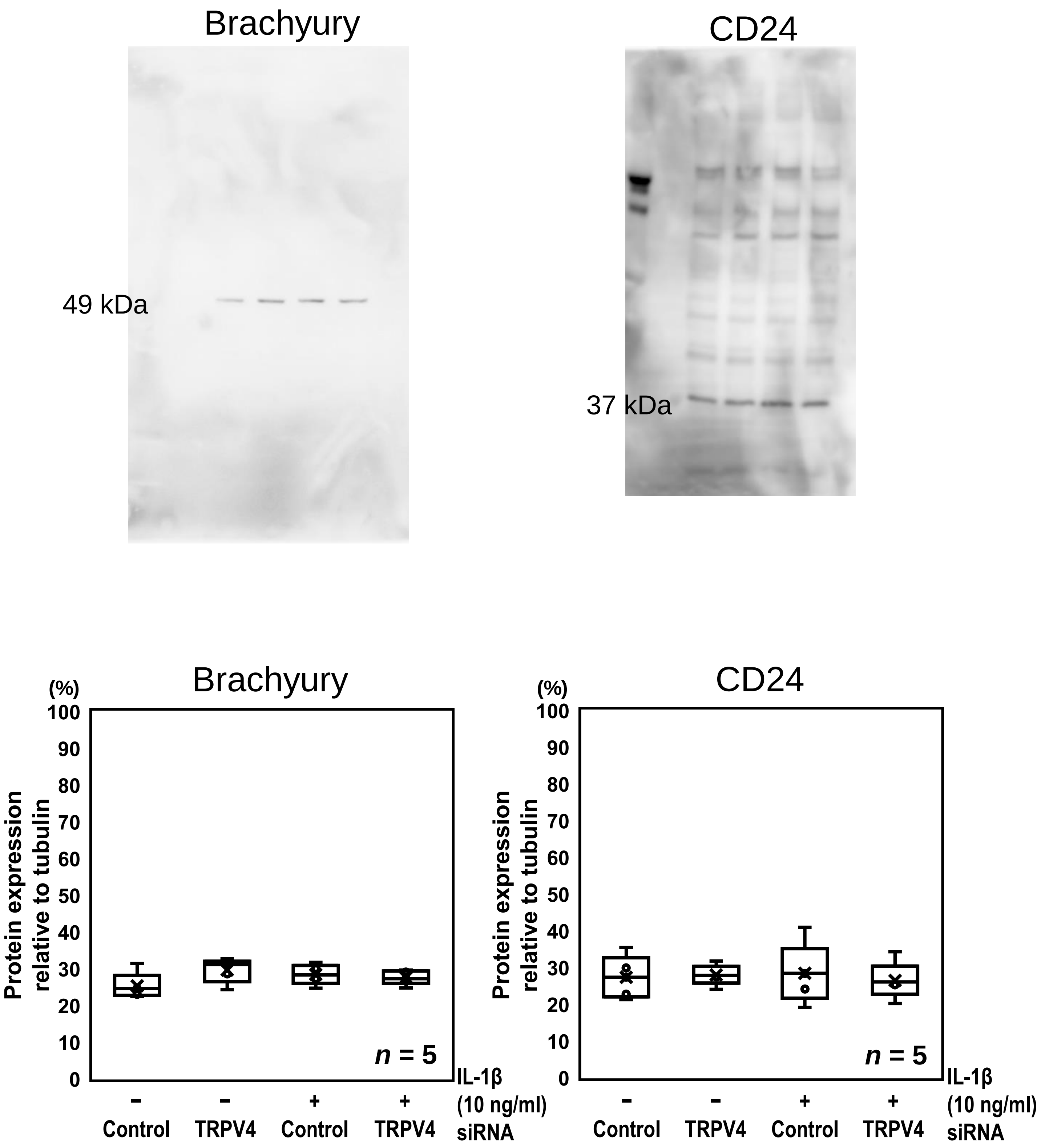

Brachyury
49 kDa
CD24
37 kDa
Brachyury
(%)
100
90
80
70
60
50
40
30
20
10
0
Protein expression relative to tubulin
n = 5
IL-1β (10 ng/ml)
– – + +
Control TRPV4 Control TRPV4
siRNA
CD24
(%)
100
90
80
70
60
50
40
30
20
10
0
Protein expression relative to tubulin
n = 5
IL-1β (10 ng/ml)
– – + +
Control TRPV4 Control TRPV4
siRNA

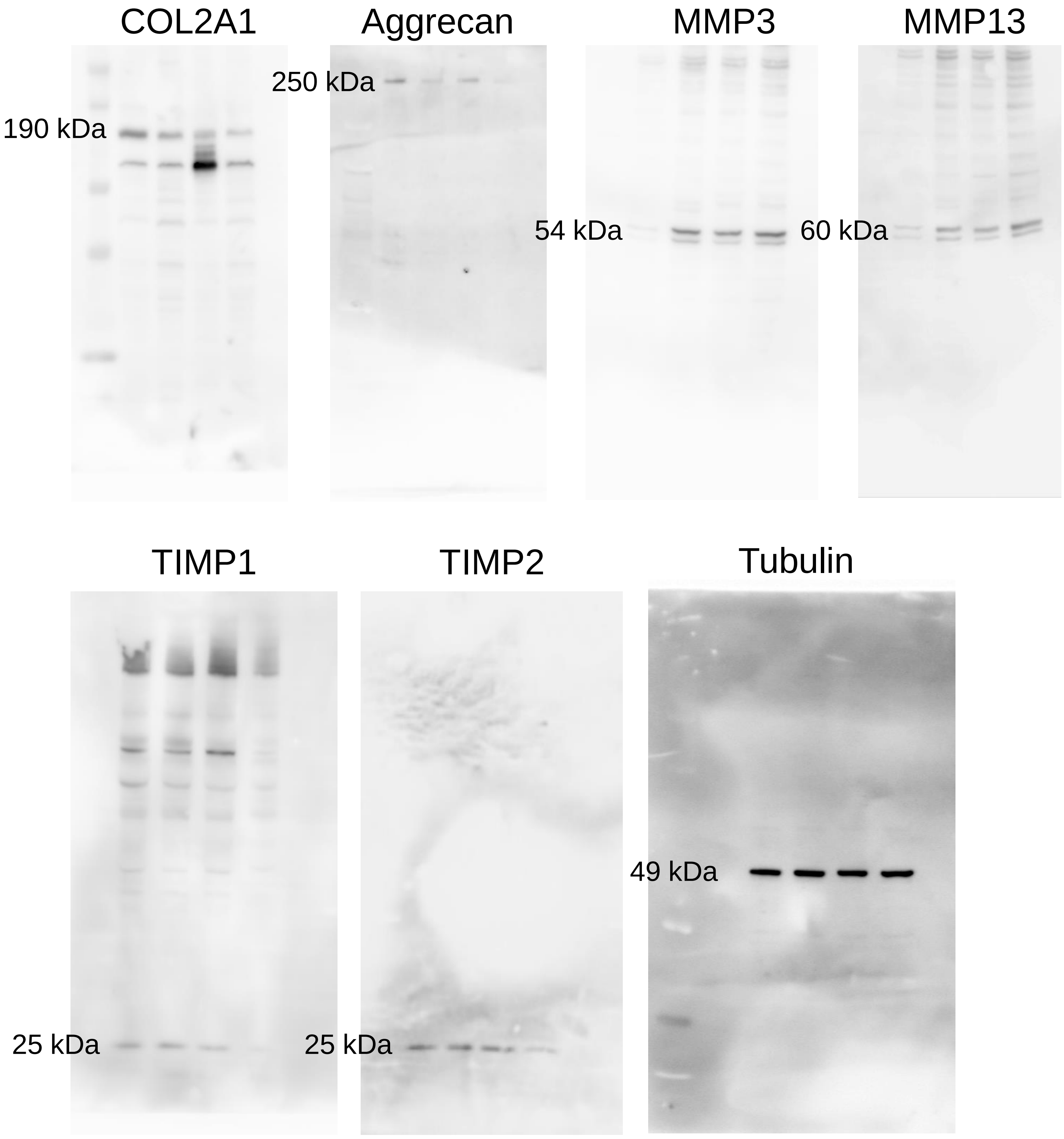
COL2A1
Aggrecan
MMP3
MMP13
190 kDa
250 kDa
54 kDa
60 kDa
TIMP1
TIMP2
Tubulin
49 kDa
25 kDa
25 kDa

Figure 4A

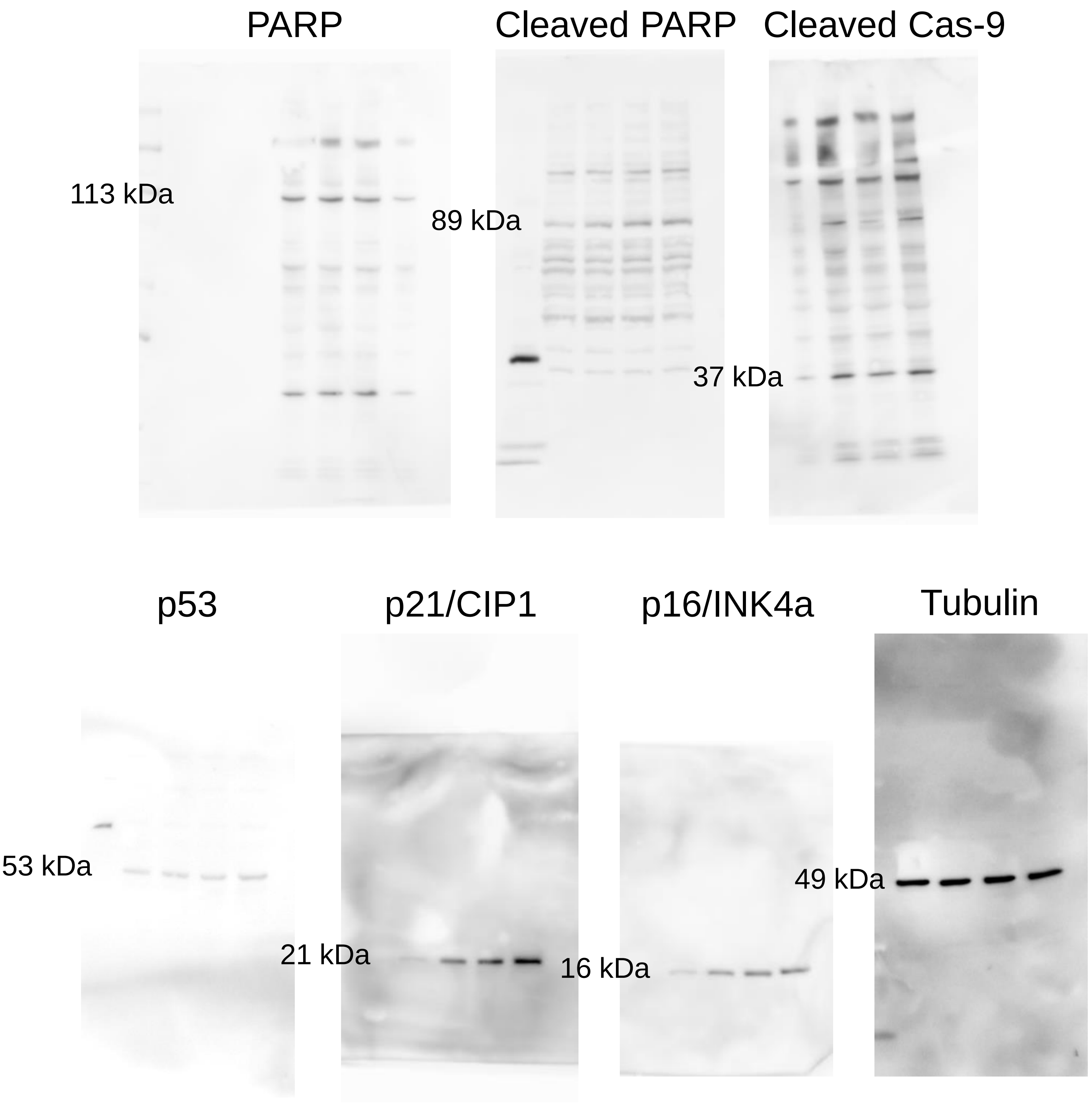
PARP
Cleaved PARP
Cleaved Cas-9
113 kDa
89 kDa
37 kDa
p53
p21/CIP1
p16/INK4a
Tubulin
53 kDa
21 kDa
16 kDa
49 kDa

Figure 5A

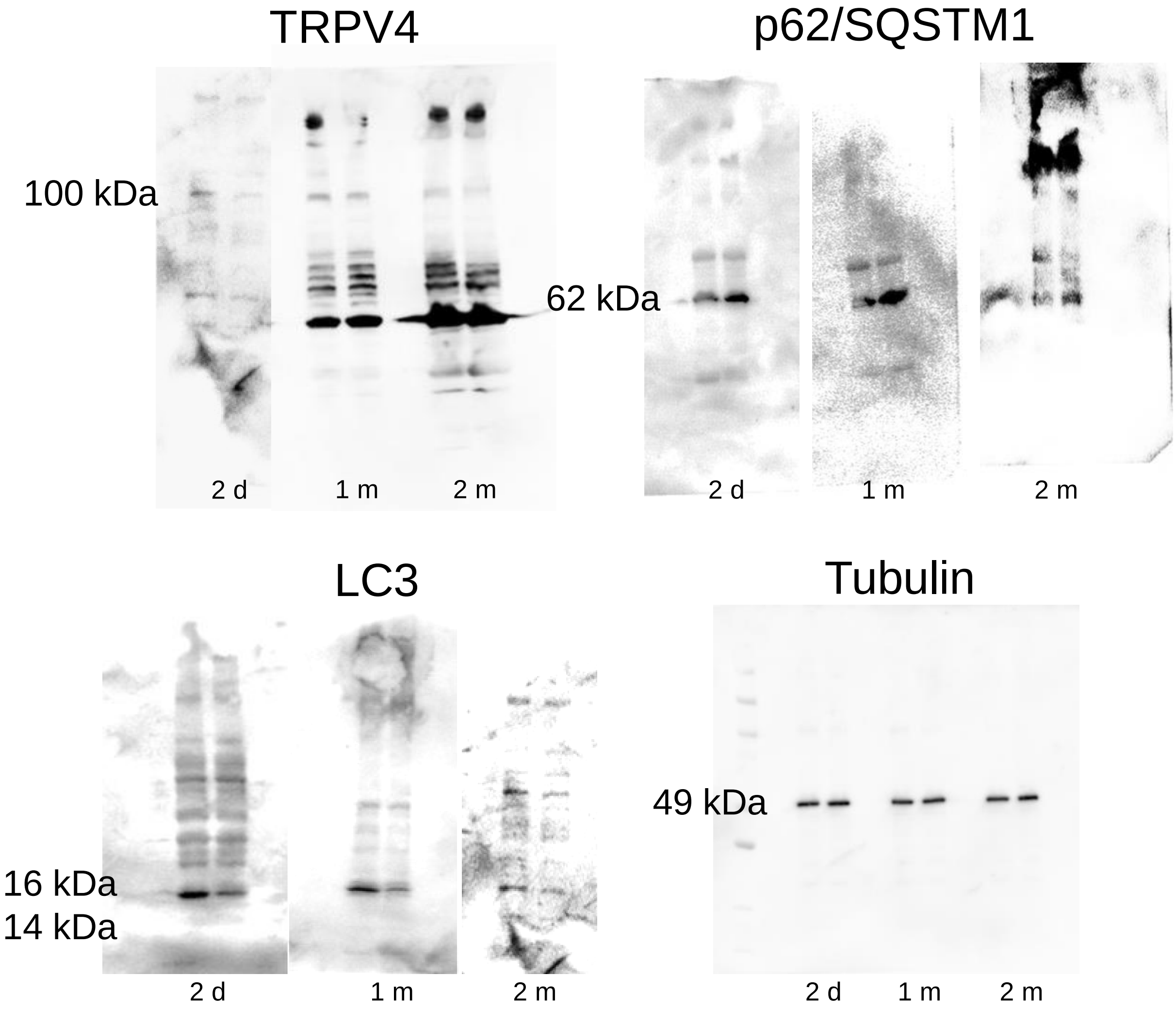
TRPV4
p62/SQSTM1
100 kDa
62 kDa
2 d
1 m
2 m
2 d
1 m
2 m
LC3
Tubulin
16 kDa
14 kDa
49 kDa
2 d
1 m
2 m
2 d
1 m
2 m
